# A Computationally Efficient Method to Generate Plausible Conformers for Ensemble Docking and Binding Free Energy Calculations

**DOI:** 10.1021/acs.jcim.5c00431

**Published:** 2025-07-23

**Authors:** Ö. Zeynep Güner Yılmaz, Pemra Doruker, Ozge Kurkcuoglu

**Affiliations:** † Department of Chemical Engineering, 52971 Istanbul Technical University, Istanbul 34467, Turkey; ‡ Department of Computational and Systems Biology, School of Medicine, 12317 University of Pittsburgh, Pittsburgh, Pennsylvania 15213, United States

## Abstract

This study presents a computationally efficient approach to generate plausible protein conformers for ensemble docking to enable evaluations of interactions between ligand and protein for ranking the docked ligands according to their binding affinities. Two binding regions of triose phosphate isomerase (TIM), its catalytic site with DHAP ( TIM), and its dimer interface with 3PG (TIM) involving flexible loops were investigated as case studies. The binding sites of the apo and holo forms were modeled at the atomistic scale (high resolution) while the remaining structure was coarse-grained (low resolution) leading to a mixed-resolution description of the protein. The slowest three normal modes related to the functional dynamics of TIM were obtained using the Anisotropic Network Model and employed to derive 36 conformers of the truncated high-resolution regions by assessing six deformation parameters in both directions of the harmonic motions. Through energy minimization and docking calculations in Glide, optimal extents of deformation were identified. The docked truncated structures were then subjected to independent molecular dynamics (MD) simulations to confirm the interactions of the ligands in the binding sites. To prevent the disintegration of the truncated structure, different buffer zones and harmonic restraints were assessed to finally decide on four distinct zones with restraints of 0, 25, 35, and 50 kcal/mol·Å^2^. Each conformer underwent 900 ns-long simulations across three replicates reaching a total simulation time of 15.2 μs. Binding free energy calculations were conducted using the MM-GBSA approach using the first 50, first 100, first 200, and 300 ns intervals, which pointed out that 100 ns-long simulations were sufficient to estimate the binding affinities for TIM. Results consistently indicated comparable binding energies between the intact and truncated TIM structures underscoring the approach’s reliability, where the truncated conformers also offered varying binding site geometries yielding favorable interactions. Comparative docking at the dimer interface of *and* TIM further highlighted species-specific binding dynamics, affirming the methodology’s applicability for diverse biological questions and establishing a computationally efficient approach to estimate binding free energy values even for supramolecular assemblages.

## Graphical abstract

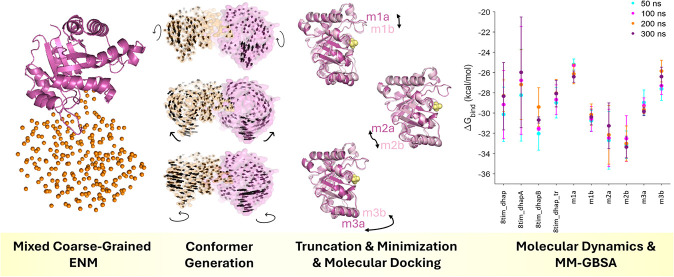

## Introduction

Accurate and rapid calculation of binding free energy in protein–ligand complexes plays a key role in structure-based drug design studies to select the hit compounds from millions of compounds and further assess with *in* *vitro* and *in* *vivo* assays. Physics-based methods, such as free energy perturbation,^1,2^ thermodynamic integration,^3^ have been applied to fragment elaboration, scaffold hopping, covalent inhibition, and binding mode validation, often achieving mean errors in the range of 1–2 kcal/mol.^4,5^ The end-point methods molecular mechanics/Poisson–Boltzmann surface area, and molecular mechanics/generalized Born surface area, allow fast scoring of large ligand sets or protein conformers with relatively modest computational resources, and have been widely used to estimate binding free energies in virtual screening or for comparative analysis.^5^ However, there exist limitations such as conformational sampling difficulties, computational costs, and the exclusion of the entropic effects in most of the cases.^6^

Depending on the availability of the data, structure-based drug design can be limited to a single protein structure in an apo or holo form that relates to a specific conformational state. Computational studies have shown that ligands select the conformers that best represent the protein’s structural and dynamic properties from those naturally accessible in the unliganded form.^7–9^ Although flexible docking methods create different conformations by leaving the protein and ligand side chains flexible, these techniques remain insufficient in fully exploring protein structures. Therefore, it is highly important to consider different conformational states of the targeted protein, besides the flexibility of the binding site, to design an effective drug. However, the lack of experimental structures providing different conformational states of the target protein is an important limitation.

Ensemble docking is the use of multiple protein conformations that can be obtained through molecular dynamics (MD) simulations in docking processes. This method has become increasingly common in drug discovery processes by providing the opportunity to evaluate protein–ligand interactions with a wider conformational diversity.^10^ Examining different conformational states of protein–ligand interactions and model binding processes is also possible for long-time scales with Markov state models.^11^ Enhanced sampling techniques,^12^ such as replica exchange MD^13^ and accelerated MD^14^ can be employed to improve conformational sampling for docking applications. They allow for more efficient modeling of dissociation processes^11^ by overcoming energy barriers. However, proper handling of conformational diversity remains a challenge,^5^ and a thorough exploration of the conformational space can be computationally intensive, especially for large or highly flexible systems.

While scanning the conformational space is easier in small protein structures, the challenge is especially evident for large complex systems. To overcome these limitations, coarse-grained (CG) models offer a simpler representation of biomolecules by reducing the number of atoms considered in simulations.^15^ CG models, such as the MARTINI model, improve protein dynamics and hydration effects while reducing computational costs.^16,17^ It can obtain unbiased dynamics of protein–ligand systems, revealing their binding-unbinding events,^18^ where backmapping is necessary to understand the details of atom interactions at the ligand-binding site. Hybrid resolution modeling approaches in MD simulations have been proposed to model the protein in mixed resolution, where the buried active site is described at atomic resolution and the remainder of the structure is CG.^19^ Similarly, PRIMO^20^ and MARTINI-based all-atom/CG schemes^21^ have been developed to balance computational cost with low structural detail, enabling simulations of large biomolecular assemblies like membrane–protein complexes or multidomain systems. These methods allow simultaneous treatment of different molecular regions at distinct levels of resolution, typically applying CG models to the environment and atomic detail to critical subunits. While effective for studying global conformational dynamics or membrane embedding, they typically require full-system parametrization or parameter tuning to fully optimize all-atom and CG region interactions.^20^ On the other hand, drug design efforts are currently focused on RNA and its complexes, where correct parametrization of protein-RNA and RNA–RNA interactions in the force fields is highly critical.^22^ Moreover, when systems as large as the rRNA-protein ribosomes are investigated, the computational load of atomic systems with explicit water presents significant obstacles in conformational sampling.^23^ To overcome such difficulties, elastic network modeling (ENM) can be used.^24^

The ENM provides a framework for understanding the flexibility of proteins by modeling low-frequency and large-scale motions.^25^ The Gaussian Network Model (GNM)^26^ is used to understand the general dynamic properties of proteins by predicting isotropic motions, while the Anisotropic Network Model (ANM)^27^ captures the specific conformational changes by examining directional motions. ANM can be used in applications such as efficient sampling of conformational space and molecular docking.^28,29^ For example, Meireles et al. showed that the smooth motion modes predicted by ANM facilitate ligand binding by allowing proteins to adopt favorable conformations that increase binding affinity.^30^ Eyal et al. highlighted that the low-frequency modes predicted by ANM are useful not only for equilibrium motions but also for efficient sampling of conformations during molecular docking calculations.^31^ The ClustENM tool analyzes different conformational states of proteins using ANM modes and facilitates the selection of the correct conformers for drug design.^32,33^ As another computationally efficient approach, a conformer selection with the Metropolis Monte Carlo algorithm among the conformations generated with ANM can be achieved, and the conformer can be further investigated with MD simulations and used to define collective motions.^34^

To address the computational limitations faced in investigating large protein complexes, we introduce a new approach to speed up the binding free energy calculations for ligand-protein complexes while providing a conformational diversity of the protein based on an available structure, either apo or holo. The novelty of the approach lies in merging ANM, truncation-based modeling,^35,36^ ligand docking, and all-atom MD simulations to calculate binding free energy values. The method generates mixed-resolution protein conformers with a binding site described at atomistic resolution, reduces the system size by truncating the all-atom binding site. Then, molecular docking calculations are performed using these conformers with an ensemble docking approach, and the binding free energies of ligands are calculated at the truncated region of the protein. The generated conformers are suitable for interaction mapping and ligand ranking without the need for full-protein modeling. The method is particularly advantageous in drug design workflows focused on pharmacophore analysis, mimicry of known ligands, or evaluation of docking poses involving the characterization of the nature of the ligand-binding site interactions for all protein complexes, including supramolecular assemblages such as the bacterial ribosome, where all-atom MD simulations and binding free energy calculations are highly expensive.

While the motivation of the study is to implement the method in drug design studies on supramolecular structures such as the bacterial ribosome, we employ the triose phosphate isomerase (TIM) enzyme of ∼500 amino acids as a model protein to demonstrate the accuracy of our method by assessing the findings using the intact protein structure. TIM is a highly conserved glycolytic enzyme that catalyzes the isomerization reaction between dihydroxyacetone phosphate (DHAP) and glyceraldehyde-3-phosphate.^37,38^ Its high catalytic efficiency is based on the dynamics of its functional regions, especially loop 6 and the homodimer interface.^39^ These regions play key roles in substrate binding and product release,^40^ and the dynamic structure of TIM has been studied using various computational methods, including ENM.^41^ We considered two different ligand binding sites on TIM of and , including the catalytic site with the functional loop 6 and the dimer interface, respectively. These regions were modeled in full atomic detail, while the rest of the structure was CG, obtaining a mixed CG (MCG) structure to reduce computational costs as compared to all-atom normal-mode analysis.^42^ ANM generated plausible conformers of the binding sites by capturing protein flexibility at the low-frequency global motions. We docked DHAP to the *Gg*TIM catalytic site and the inhibitor 3-phosphoglycerate to the *Pf*TIM dimer interface to their binding sites on different conformers using Schrödinger Glide.^43^ Then, a total of 900 ns of independent MD simulations for both apo and ligand-docked systems were performed in Desmond^44^ to assess the docking poses of the ligands. To prevent the disintegration of the truncated conformers comprising the binding sites, the structure was divided into three or four zones, and different harmonic restraints were implemented to find the optimum number of zones and strengths that maintained the conformational flexibility of the investigated site. Then, MD simulations were used to estimate the binding free energies of the ligands docked to different conformers. The calculations also showed that the optimum duration of MD simulations is sufficient to predict the binding affinity accurately for TIM. In addition, the new method revealed the structural differences between the dimer interfaces of the two species, where the interactions of the inhibitor 3-phosphoglycerate with *Pf*TIM and *Gg*TIM dimer interfaces differed. These findings indicated that the integration of the MCG ANM with the usage of a truncated region in the MD simulations can successfully predict the protein–ligand interactions, where the computational costs for a large protein are expected to be very high with the usage of all-atom normal-mode analysis and other hybrid approaches requiring the intact structure. This approach has a high potential for a broad application in computational biology and drug discovery, especially for supramolecular assemblages such as the antibiotic target ribosome.

## Materials and Methods

### Mixed Resolution Anisotropic Network Model

The protein structure can be described at a mixed-resolution to perform atomistic modeling in the functional regions of the protein, and a lower-resolution modeling is applied in the remaining parts to reduce the computational cost of obtaining its functional dynamics.^42^ To construct the mixed coarse-grained (MCG) elastic network, the heavy atoms are taken as the nodes for the so-called high-resolution (HR) region, and the remaining parts are modeled at a one-node-per-residue level with the nodes placed at the Cα atom or center of mass of the residues to form the low-resolution (LR) region. The neighboring nodes are determined based on a single atomic cutoff distance of 10 Å, where a cutoff value of 6 to 10 Å between heavy atoms was demonstrated as suitable to obtain the functional dynamics.^42,45^ The spring constant γ_*ij*_ changes according to pairwise interactions of *i*th and *j*th residues, and it is calculated based on their number of heavy atom—heavy atom contacts within the cutoff distance. Potential energy is defined as,

1 $$V=\underset{i}{\sum }\underset{j}{\sum }\frac{\gamma_{ij}}{2}h\left(r_{\mathrm{cut}}-R_{ij}\right){\left(\Delta R_{j}-\Delta R_{i}\right)}^{2}$$

where **R**_*i*_ and Δ**R**_*i*_ indicate the position and fluctuation vectors of node *i* (1 < *i* ≤ *N*, where *N* is the number of nodes), while *R*_*ij*_ represents the distance between node pairings (*i*, *j*). The function *h*(*x*) represents the Heaviside step function, which returns 1 when *x* ≥ 0, indicating that the nodes are close enough to interact, and 0 otherwise.

By using the MCG approach, a spherical region of radius 30 Å centering the ligand was selected and modeled at HR. This region enclosed the functional sites of the protein, namely the catalytic domain with loop 6 of TIM (PDB ID: 8TIM and PDB ID: 1TPH) and the dimer interface region of TIM (PDB ID: 1VGA and PDB ID: 2VFI). The rest of the protein structure was represented at LR in both cases. Missing loops and short segments in the structures were completed during the modeling process, allowing the construction of the MCG structure. The HR region focusing on the catalytic site accommodated most of the subunit B in *Gg*TIM; therefore, the whole subunit B was considered at atomic resolution. The size of the HR region was considered suitable for the truncation-based simulations, where similar studies have adopted truncation radii in the range of 15–40 Å.^46–49^ In addition, the size of the HR region was large enough to enclose an inner region with a radius of 15 Å around the ligand that was kept flexible during the energy minimization and the molecular dynamics simulations.

The structure obtained with MCG was used to construct the Hessian matrix **H**, which was mass-weighted as **M** ^–1/2^ **HM** ^–1/2^ using the node (residue) masses stored in the matrix **M**. Based on the mass-weighted Hessian, the normal-mode analysis was employed to calculate the eigenvalues **Λ** and eigenvectors **U** and to obtain 3*N*-6 distinct vibrational motions, where six modes correspond to the rigid body translational and rotational motions. The residue fluctuations $\left\langle \Delta R_{i}^{2}\right\rangle$ can be calculated over all vibrational modes as,

2 $$\left\langle \Delta R_{i}^{2}\right\rangle =\overset{3N-6}{\underset{k=1}{\sum }}\frac{u_{ik}^{2}}{\lambda_{k}}$$

where $u_{ik}^{2}$ and λ_*k*_ is the magnitude of residue displacement, and eigenvalue of the *k* ^th^ mode, respectively.

In this study, the slowest three modes were considered for the analysis of the globular motions of the enzyme, which were previously shown to correspond to its functional motions.^45,50^ Two conformers from each mode were obtained due to the harmonic motion, thus producing a total of six conformers. These conformers were named “mXa” (positive) and “mXb” (negative) to indicate the direction of motion, where “X” represents the mode number (1, 2, or 3). The conformational flexibility of the protein structure was tested using six different deformation parameters exaggerating the motions, each resulting in root-mean-square deviations (RMSD) of 1.0, 1.5, 2.0, 2.5, 3.0, and 4.0 Å based on the Cα atoms when compared to the native structure. RMSD was calculated as

3 $$\mathrm{RMSD}=\sqrt{\frac{1}{N}\overset{N}{\underset{i=1}{\sum }}{\left(r_{i}-r_{r}^{\mathrm{ref}}\right)}^{2}}$$

Here, *N* represents the number of atoms in the structure being compared based on Cartesian coordinates *r*. *r* ^ref^ represents the atom coordinates in the reference structure.

As a result, a total of 36 different conformers were obtained.

### Energy Minimization

The HR regions of each MCG conformer generated with different deformation parameters were extracted from the intact structure. Similarly, for the native structure, the residues that were fully atomic in the MCG model were selected and extracted. These truncated regions were then subjected to energy minimization using NAMD v1.9 program^51^ with the CHARMM 36 force field parameters.^52^ The Generalized Born Implicit Solvent model was used with a 12 Å Born radius and 0.3 M ion concentration. The time step was set to 2 fs, and constant temperature control was provided using Langevin dynamics. Energy minimization was performed with the convergence criterion 0.001 kcal/mol. The inner region with a radius of 15 Å, where the docking calculations were carried out, was kept flexible. An external force of 50 kcal/mol·Å^2^ was applied to the atoms with a distance more than 15 Å from the ligand, to maintain the structural integrity of the structures and prevent them from disintegrating, as in a previous study.^35^

### Molecular Docking Studies

The molecular docking calculations were performed for both the catalytic region of *Gg*TIM (PDB ID: 8TIM and PDB ID: 1TPH) and the interface region of *Pf*TIM (PDB ID: 1VGA and PDB ID: 2VFI). The ligand phospho glycolohydroxamate (PGH) is accommodated at the catalytic site of the *Gg*TIM crystal structure (PDB ID: 1TPH). The ligand 3-phosphoglycerate (3PG) is located at the dimer interface of *Pf*TIM (PDB ID: 2VFI). The other crystal structures are in apo form. First, validation docking was performed on holo-structures using Schrödinger Glide^43^ software by reproducing the crystal poses of the native ligands and their interactions in these regions. Proteins were prepared with Protein Preparation Wizard at pH 7 ± 0.5, hydrogen atoms were added, and heteroatoms were created. In addition, hydrogen bonds were optimized at pH 7 using PROPKA. The LigPrep module was used in the preparation of ligands at pH 7 ± 0.5. Docking was performed with the Extra Precision (XP) module and a maximum of 10 poses was created for each ligand. After the validation docking process for the catalytic site of *Gg*TIM, the intermediate ligand PGH was replaced with the substrate dihydroxyacetone phosphate (DHAP) using molecular docking. The validated docking procedure was applied to the energetically minimized full atomic regions of MCG structures with different deformations. For comparison, docking was performed on both chains of the intact apo homodimers. After XP-docking, flexible Prime MM-GBSA calculations were performed by starting with the energy minimization of the receptor and ligand separately using the OPLS2005 force field^53^ and the VSGB 2.0 solvation model, then the energy minimization of the receptor–ligand complex and the energy calculations of the receptor and ligand of the minimized complex according to the following equation:

4 $$\Delta G_{\mathrm{bind},\mathrm{Prime}}=E_{\mathrm{complex}}^{\mathrm{minimized}}-(E_{\mathrm{receptor}}^{\mathrm{minimized}}+E_{\mathrm{ligand}}^{\mathrm{minimized}})$$

### Molecular Dynamics Simulations

Plausible protein–ligand complexes obtained from molecular docking studies were further investigated through molecular dynamics (MD) simulations, using poses from Prime calculations as the initial structures. The Schrödinger Desmond software package^44^ was employed for the simulations, utilizing the OPLS2005 force field. The protein–ligand complexes were solvated in an orthorhombic box shape with a buffer width of 10 Å, employing the TIP3P water model to mimic the solvent environment. The system was neutralized by adding sodium ions, and a salt concentration of 0.15 M NaCl was adopted. The minimization was performed for 5 ns to relax the system. Subsequently, the five-step relaxation protocol of Desmond was executed. First, the system was simulated in the NVT ensemble using Brownian dynamics at 10 K with small time steps, while restraining non-hydrogen solute atoms. This was followed by simulation in the NVT ensemble with the Berendsen thermostat^54^ at 10 K for 12 ps, applying fast temperature relaxation and velocity resampling every 1 ps, still restraining non-hydrogen solute atoms. Next, the simulation was conducted in the NPT ensemble with the Berendsen thermostat and barostat,^54^ maintaining a temperature of 10 K and a pressure of 1 atm for 12 ps, with fast temperature relaxation and slow pressure relaxation, alongside velocity resampling every 1 ps and non-hydrogen solute atom restraints. The procedure continued with simulations at 300 K and 1 atm pressure for 12 ps, with fast temperature relaxation and slow pressure relaxation, still restraining non-hydrogen solute atoms. Finally, a 24 ps simulation in the NPT ensemble was performed at 300 K and 1 atm pressure, with fast temperature relaxation and normal pressure relaxation, while restraining non-hydrogen solute atoms. Subsequently, two or three independent replicas of MD simulations with 2 fs time step were carried out for 20 different systems as detailed in Table S1, reaching a total simulation time of 15.2 μs. Electrostatic interactions were calculated using the Particle Mesh Ewald method with a cutoff of 12 Å.

The MD trajectories were subjected to Molecular Mechanics Generalized Born Surface Area (MM-GBSA) calculations using Schrödinger’s thermal MM-GBSA Python script. This step allowed for estimating the binding free energy ΔG_bind_ and provided insights into the stability and strength of protein–ligand interactions throughout the simulations. The contribution of entropy was neglected. Calculations were performed for the full simulation trajectory of 300 ns and the first 200, 100, and 50 ns, allowing comparison of binding free energy values at different periods. Calculations were carried out over all frames and recorded every 600 ps for each period. Furthermore, Principal Component Analysis (PCA) was performed using ProDy to calculate the first 10 PCs to analyze the dynamic motions and conformational changes observed in the MD trajectories.^55,56^

### Conformer Generation Using AlphaFold2

The AlphaFold2 (AF2) was used to produce native-like conformers of *Gg*TIM through the AlphaFold Protein Structure Database server (https://alphafoldserver.com) and ColabFold,^57^ which is a faster and more accessible version of AF2 optimized for local and cloud-based execution. Twelve conformers were generated with the AF2 server, including both monomeric and dimeric structures of TIM, where the dimer is the functional form. Four different settings were tried, with three different random seeds to obtain different conformers: (1) dimer with template (called af1-af3), (2) dimer without template (af4-af6), (3) monomer with template (af7-af9), and (4) monomer without template (af10-af12). The AF2 server automatically selected structural templates when enabled. The ColabFold notebook can generate monomeric structures using different multiple sequence alignment (MSA) settings. In the default configuration, MMSeqs2 was used to produce full MSAs with random seeds to encourage conformational diversity. Then, MSA depth was controlled to obtain distinct native-like conformers by testing a reduced-depth MSA setup (max_msa_clusters = 16 and max_extra_msa = 32), and a high-depth MSA setup (max_msa_clusters = 64 and max_extra_msa = 128). In total, six representative ColabFold monomeric conformers (called cf) were selected across these setups: cf1 from default (auto MSA, rank 1), cf2–cf4 from reduced MSA (ranks 1, 2, and 3), and cf5–cf6 from high-depth MSA (ranks 1 and 5).

Two confidence metrics of AF2, predicted Local Distance Difference Test (pLDDT) and predicted template modeling score (pTM) were employed to assess the reliability of 18 AF2 models. The pLDDT score provides per-residue confidence values ranging from 0 to 100, and reflects the model’s expected local structural accuracy. It is based on the local Distance Difference Test for Cα atoms (lDDT-Cα), which estimates how well the predicted Cα–Cα distances match the true structure, without requiring global superposition.^58,59^ In ColabFold, the average pLDDT across the structure is reported, whereas the AF2 server displays per-residue values categorized into four confidence levels: >90 (very high), 70–90 (confident), 50–70 (low), and <50 (very low). The pTM score was used to evaluate the overall structural confidence, including domain packing accuracy, in both monomeric and dimeric TIM models. The pTM score ranges from 0 to 1, with values greater than 0.8 typically indicating a reliable global topology.^60,61^ Unlike pLDDT, which provides residue-level local accuracy, pTM reflects the consistency of the predicted structure at the domain or chain level. Then, the structural heterogeneity of all *Gg*TIM conformers was evaluated by calculating root mean-squared deviation (RMSD) of loop 6 (residues Trp168–Pro178) of the conformers by taking the apo structure (PDB ID: 8TIM) as reference. In addition, Cα–Cα distances were measured between residue pairs Gly171 on the tip of loop 6 and Gly210 and Gly171 and Asn216 to reflect the degree of loop closure over the active site. For reference, these measurements were also performed on the apo structure (PDB ID: 8TIM) and the holo structure (PDB ID: 1TPH), displaying open and closed conformations of loop 6, respectively.

## Results and Discussion

### The Catalytic Site of *Gg*TIM

#### Conformer Generation Using Mixed Coarse-Graining

We used a mixed coarse-grained (MCG) description^42^ of the apo *Gg*TIM structure (PDB ID: 8TIM) focusing on its catalytic site and the functional loop 6 in an open conformation. The catalytic residues with which the ligand interacts are Lys13, His95, and Glu165. A spherical region of 30 Å radius centering the catalytic site was selected and represented in high resolution (HR), i.e., including the heavy atoms. As the HR region included most of the subunit B, the whole subunit was considered in full atom. The remaining residues, i.e., subunit A, were described in low resolution (LR) as one-node-per-residue. *Gg*TIM structure is presented in Figure 1a with the MCG representation.

**Figure 1:**
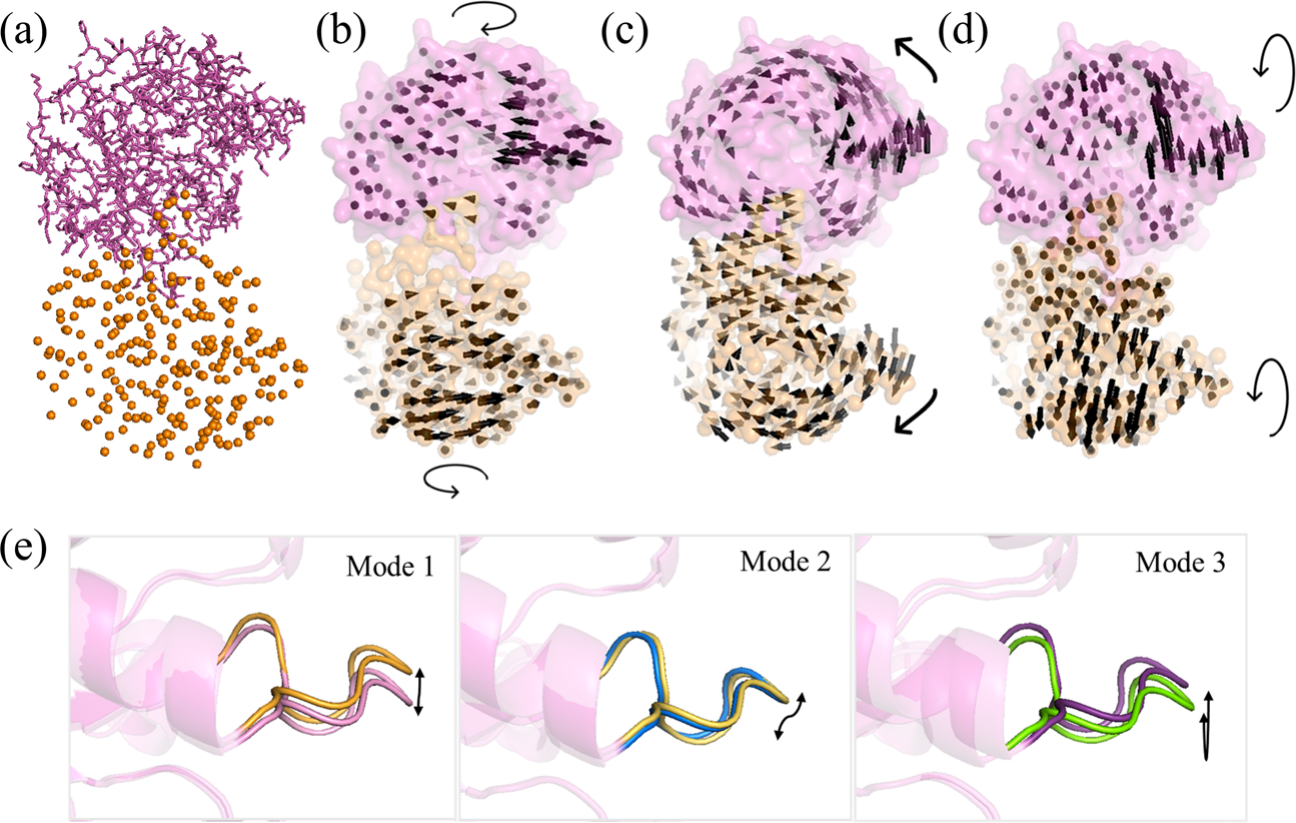
(a) Mixed-resolution structure of *Gg*TIM, incorporating both HR (subunit B, in pink sticks) and LR (subunit A, in orange spheres) regions. Visualization of (b) the first, (c) second, and (d) third slowest modes calculated for the MCG structure. (e) Dynamic behavior of loop 6 in the slowest modes, highlighting its opening and closing motions. For clarity, the residue displacements in panels b to d are exaggerated and only shown for Cα atoms using arrows.

The residue displacements were calculated with the Anisotropic Network Model (ANM) using the normal-mode analysis for the low-frequency end of the spectrum, and distinct protein conformations were generated. The model’s accuracy in capturing the MCG structure’s dynamic behavior was assessed with the experimental B-factors (Figure S1), yielding a correlation coefficient of 0.67, showing a good agreement. The slowest three modes were obtained for the mixed-resolution *Gg*TIM that captures the functional motions of the protein.^45,50^ Figure 1b–d displays the harmonic motions from Mode 1, Mode 2, and Mode 3, respectively. In Mode 1, subunits A and B rotate in opposite directions as separate dynamic domains. This motion indicates a twisting movement of the entire structure around an axis that passes through the core regions of the TIM barrels. Mode 2, on the other hand, highlights the bending of the subunits around the dimer interface, acting as a hinge domain. Mode 3 showcases another bending-type motion.^62^

Upon examining the dynamics of loop 6 in these modes, distinct conformational changes emerge, offering insights into the extent of its flexibility (Figure 1e). In the first mode, loop 6 exhibits a characteristic opening and closing motion over the catalytic site. In the second mode, it demonstrates a lateral movement, like swaying from side to side. A combination of lateral and transverse motions is observed in the third mode. Indeed, there is a high level of overlap when comparing the low-frequency motions of loop 6 with the motions observed in crystal structures,^45^ which supports the accuracy and validity of the method. This approach enabled efficient exploration of conformational space while preserving essential structural characteristics, particularly in functionally significant regions like loop 6 of TIM.

Based on the three slowest modes, the resulting displacement vectors of the residues in HR and LR regions were exaggerated with a deformation parameter to obtain distinct protein conformers. This parameter should be selected carefully to provide sufficient flexibility for protein movements so that the structure maintains its robustness and does not experience excessive deformation or structural distortion. An appropriate deformation parameter is expected to capture the conformational changes of the protein while preserving the structural integrity to finally result in a plausible conformer. Deformations in the 1.0–2.0 Å range reflect commonly accepted physical fluctuations in ENM-based models, including TIM.^63,64^ However, the conformational flexibility of proteins depends on their contact topology and their shapes. In line with this, a previous ENM study generated binding-competent intermediates by deforming the protein structures and filtering the plausible conformers based on their energetically minimized structures.^65^ Similarly, in this study, we adopted a gradual scaling strategy to explore a broad range of conformational flexibility to yield 1.0–4.0 Å RMSD conformers and to test the system’s structural tolerance, assessed with energy minimization as in ref 65. The deformations were induced in the first three mode shapes to obtain structures with six different root-mean-square displacement (RMSD) values of 1.0, 1.5, 2.0, 2.5, 3.0, and 4.0 Å compared to the minimum structure before minimization. Figure 2a shows the energetically minimized truncated conformers, i.e., only the HR region, obtained by deforming the initial structure in the positive direction of the normal modes superimposed on the minimum structure. As the deformation scale increases, loop 6 is observed to open out more. This is consistent in both positive and negative directions, indicating a bidirectional flexibility. Figure 2b shows the structures deformed in the negative direction, with the inward movements of loop 6 toward the catalytic site becoming more pronounced as the deformation increases.

**Figure 2:**
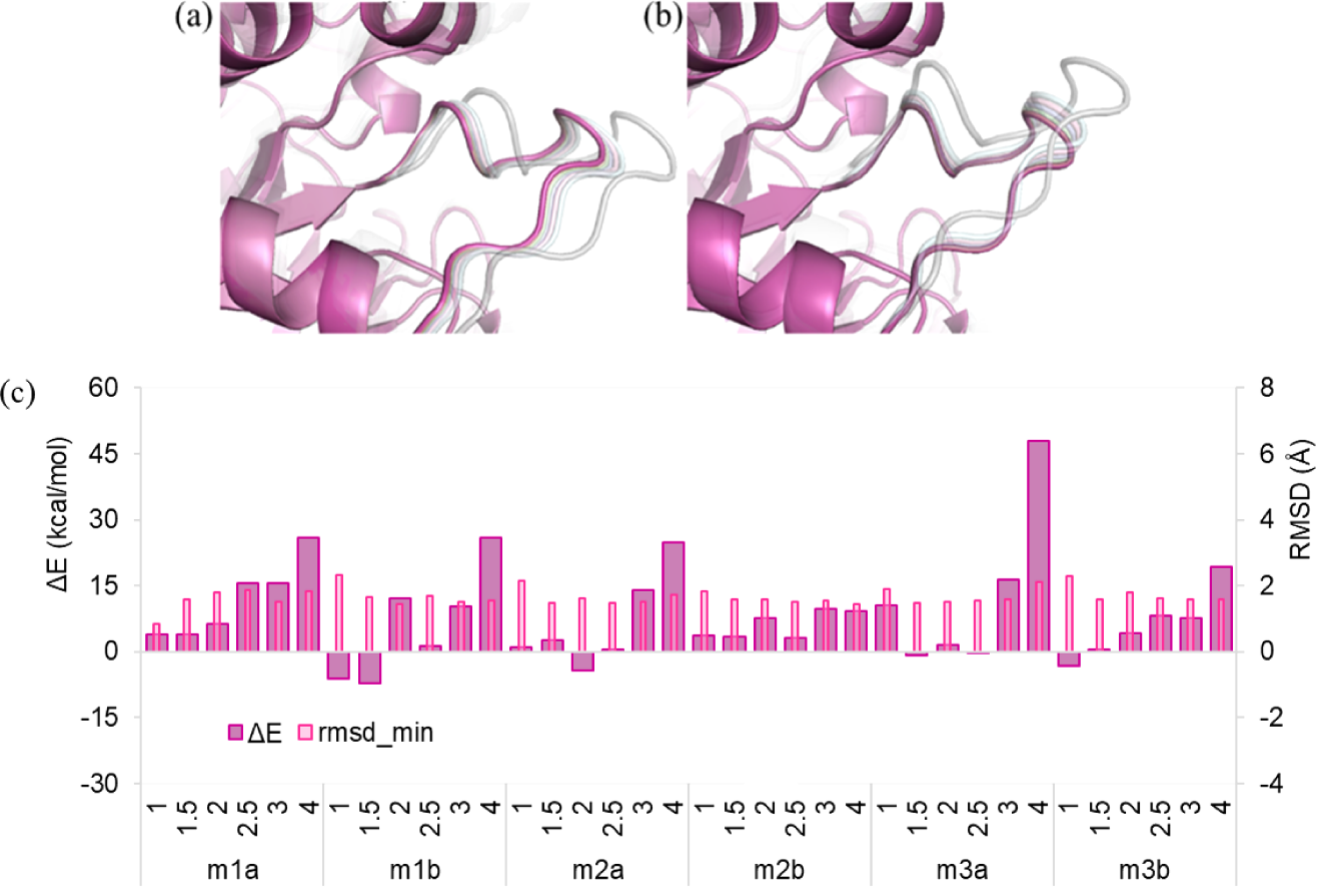
Positive (a) and negative (b) direction conformers obtained by applying different deformation parameters, resulting in RMSD values of 1.0, 1.5, 2.0, 2.5, 3.0, and 4.0 Å in both directions of the first mode. Pink and gray cartoons represent the minimum structure and the conformers, respectively. (c) Energy differences between the energetically minimized conformer and the minimum structure (Δ*E*), and postminimization RMSD values (rmsd_min) for *Gg*TIM are plotted against the deformation RMSD values (1.0–4.0 Å) for each mode (m1a-m3b), given on the x-axis.

Figure 2c compares the energy minimization results of the conformers obtained with different extents of deformation using the HR region extracted from the intact structure. The HR region for the native structure was also subjected to energy minimization to use it as a reference to select plausible conformers, similar to a previous study.^65^ A spherical region of radius 15 Å on the HR region, including the catalytic site, was not restrained, while an external force of 50 kcal/mol·Å^2^ was applied to the remaining truncated structure to prevent disintegration during the energy minimization. It is worth mentioning that the HR region corresponds to subunit B, and it is possible to minimize the energies of the generated conformers without applying restraints. However, the HR region may involve a truncated region without a continuous primary sequence, as will be illustrated for another case investigated in this study. Considering the bidirectional deformations, energy values higher than the minimum structure were observed, especially at deformations of 2.0 Å and above. For the deformations of 1.0 and 1.5 Å, the energy values of the minimized conformers were either below the energy threshold or slightly higher by a difference of ∼5 kcal/mol, except for m3a, displaying an energy value with 10 kcal/mol above the threshold (i.e., the minimum structure). Figure 2c can also be used to evaluate the sensitivity to deformation magnitude, using the difference between the energies of the deformed structure and the initial structure after minimization versus the deformation RMSD (1.0–4.0 Å). RMSD values after minimization (rmsd_min) were also computed to assess the structural deviation retained postenergy minimization. The energy differences and rmsd_min values indicated that moderate deformations (1.0–1.5 Å) generally retained structural stability with low energy penalties and limited deviation postminimization in the catalytic region, whereas larger displacements resulted in structures with high energy in a mode-dependent manner. For the investigated region of *Gg*TIM, the conformers with up to 1.5 Å seemed to preserve their structural integrity. Thus, these were considered for further molecular docking studies, which also served to assess the selected conformers. Here, it should be noted that the energy minimization can potentially bias the conformational sampling toward the initial structure, eliminating alternative ligand binding modes during the docking calculations and MD simulations. Therefore, the shape of the binding site in the generated conformers should be analyzed to ensure conformational diversity.

### Molecular Docking Calculations

The docking protocol was validated by redocking the intermediate analog phosphoglycolohydroxamate (PGH) to its binding site on *Gg*TIM (PDB ID: 1TPH). Figures S2a and S2b show the interactions of PGH in the crystal structure and after redocking, respectively. Figure S2c presents the distances of the nonbonded interactions after PGH docking. Hydrogen bonds were observed at distances of 2.27 Å (2.32 Å) with Lys13, 2.87 Å (2.71 Å) with His95, and 2.97 Å (2.91 Å) with Glu165 in subunit A (B). These distances are consistent with those reported for PGH-TIM interactions in the literature, confirming the redocking accuracy.^66^ In the validation docking with PGH to *Gg*TIM, Prime MM-GBSA energies of −29.27 kcal/mol and −33.87 kcal/mol were obtained for subunits A and B, respectively (Table 1).

**Table 1:** Prime MM-GBSA Values for the Ligands Docked to the *Gg*TIM Catalytic Cavity^a^

Structure	Ligand	RMSD before minimization^b^ (Å)	RMSD after minimization (Å)	Prime MM-GBSA (kcal/mol)
Holo *Gg*TIM (subunit A)	PGH	-	0.2	–29.27
Holo *Gg*TIM (subunit B)	PGH	-	0.2	–33.87
Holo *Gg*TIM (subunit A)	DHAP	-	0.3	–58.31
Holo *Gg*TIM (subunit B)	DHAP	-	0.1	–48.15
Apo *Gg*TIM (subunit A)	DHAP	-	0.9	–39.91
Apo *Gg*TIM (subunit B)	DHAP	-	0.9	–36.94
Apo *Gg*TIM (truncated, subunit B)	DHAP	-	0.02	–34.71
*Gg*TIM_m1a	DHAP	1.0	1.5	–7.63
*Gg*TIM_m1b	DHAP	1.0	1.2	–4.09
*Gg*TIM_m2a	DHAP	1.0	1.2	–8.34
*Gg*TIM_m2b	DHAP	1.0	0.9	–17.92
*Gg*TIM_m3a	DHAP	1.0	1.4	–8.56
*Gg*TIM_m3b	DHAP	1.0	1.2	–15.36
*Gg*TIM_m1a	DHAP	1.5	1.2	–7.82
*Gg*TIM_m1b	DHAP	1.5	1.2	–1.91
*Gg*TIM_m2a	DHAP	1.5	1.2	–18.55
*Gg*TIM_m2b	DHAP	1.5	1.2	–14.24
*Gg*TIM_m3a	DHAP	1.5	1.2	–8.16
*Gg*TIM_m3b	DHAP	1.5	1.2	+20.89

a The root mean-squared deviations (RMSD) of the conformers before and after energy minimization are also listed.

b The reference structure is apo *Gg*TIM (truncated, subunit B).

PGH was then replaced with dihydroxyacetone phosphate (DHAP), the substrate of TIM, as in a previous study.^67^ The interactions between DHAP and holo *Gg*TIM (PDB ID: 1TPH) after docking calculations are shown in Figures S3a and S3b, and the docking scores are listed in Table S2. Similar to PGH, DHAP interacts with catalytic residues by forming hydrogen bonds through His95 and Lys13. Stronger interactions in subunit A, especially with Glu165, resulted in a higher Prime MM-GBSA energy value of DHAP (−58.31 kcal/mol) (Table 1). In subunit B, this was calculated to be slightly lower (−48.15 kcal/mol) due to a lower number of favorable interactions with the residues.

In structure-based drug design, apo structures of proteins are usually employed for molecular docking calculations. DHAP was then docked to subunits A and B of apo *Gg*TIM (PDB ID: 8TIM) with loop 6 in an open conformation, revealing the catalytic cavity to the solvent. The Prime MM-GBSA energy values in the DHAP docked apo-structure (−39.91 and −36.94 kcal/mol) were lower when compared with those of the holo-structure (Table 1). This is an expected result since loop 6 is in a closed conformation in the holo-structure, leading to a more confined space that increases the extent of nonbonded interactions of the ligand with the binding site. Interactions after the docking calculations are presented in Figure S3b. Key interactions include Lys13 forming two salt bridges with oxygens of the phosphate group, Asn11 forming hydrogen bonds with the hydroxyl group, and His95 interacting with both the hydroxyl group on subunit A and =O on subunit B. These interactions agree with the literature,^68,69^ confirming the accuracy of the docking calculations with DHAP. Then, the docking calculations were performed for the truncated apo *Gg*TIM with loop 6 in open conformation, and the Prime MM-GBSA energy was found as −34.71 kcal/mol, similar to the docking results with the intact apo *Gg*TIM. In the truncated structure, the hydrogen bond with Asn11 observed in the intact structure is lost, which can explain the slightly better Prime MM-GBSA energy observed in the intact B chain.

Among the generated conformers for the three slowest modes, structures with deformation of 1.0 Å and 1.5 Å were evaluated for molecular docking. The RMSD values of the deformed structures after the minimization changed slightly (Table 1). For the conformers with a 1.0 Å deformation before minimization, Prime MM-GBSA values were between −17.92 and −4.09 kcal/mol, whereas this range was from −18.55 to +20.89 kcal/mol for the conformers with deformation of 1.5 Å, although their RMSD values after minimization were 1.2 Å (Table 1). Here, the unfavorable binding energy (+20.89 kcal/mol) was observed for the m3b conformation. This is due to the ligand not providing sufficient stability in the binding site, and unfavorable interactions. TIM conformers deformed with high RMSD values above 2.0 Å were also tested in the docking calculations, but generally yielded scores with positive values (not shown). Therefore, the conformers with the extent of deformation 1.5 Å and higher were eliminated. Among the conformers with deformation of 1.0 Å, the m2b and m3b conformers exhibited the best Prime MM-GBSA values (−17.92 kcal/mol and −15.36 kcal/mol, respectively). DHAP displayed higher Prime MM-GBSA values for these conformers by establishing interactions with critical residues such as Asn11, Lys13, and Glu165. The other conformers showed lower Prime MM-GBSA values. The interactions between docked DHAP and apo *Gg*TIM conformers and the distances of key residues for each of the six conformations obtained with a deformation RMSD of 1.0 Å are also displayed in Figure 3. This diversity allowed us to analyze the effects of the conformers on the flexibility and functional dynamics of the protein on ligand binding. The different conformers showed a slightly different binding pose of the ligand, reflecting the flexibility of the catalytic cavity due to the protein’s functional dynamics.

**Figure 3:**
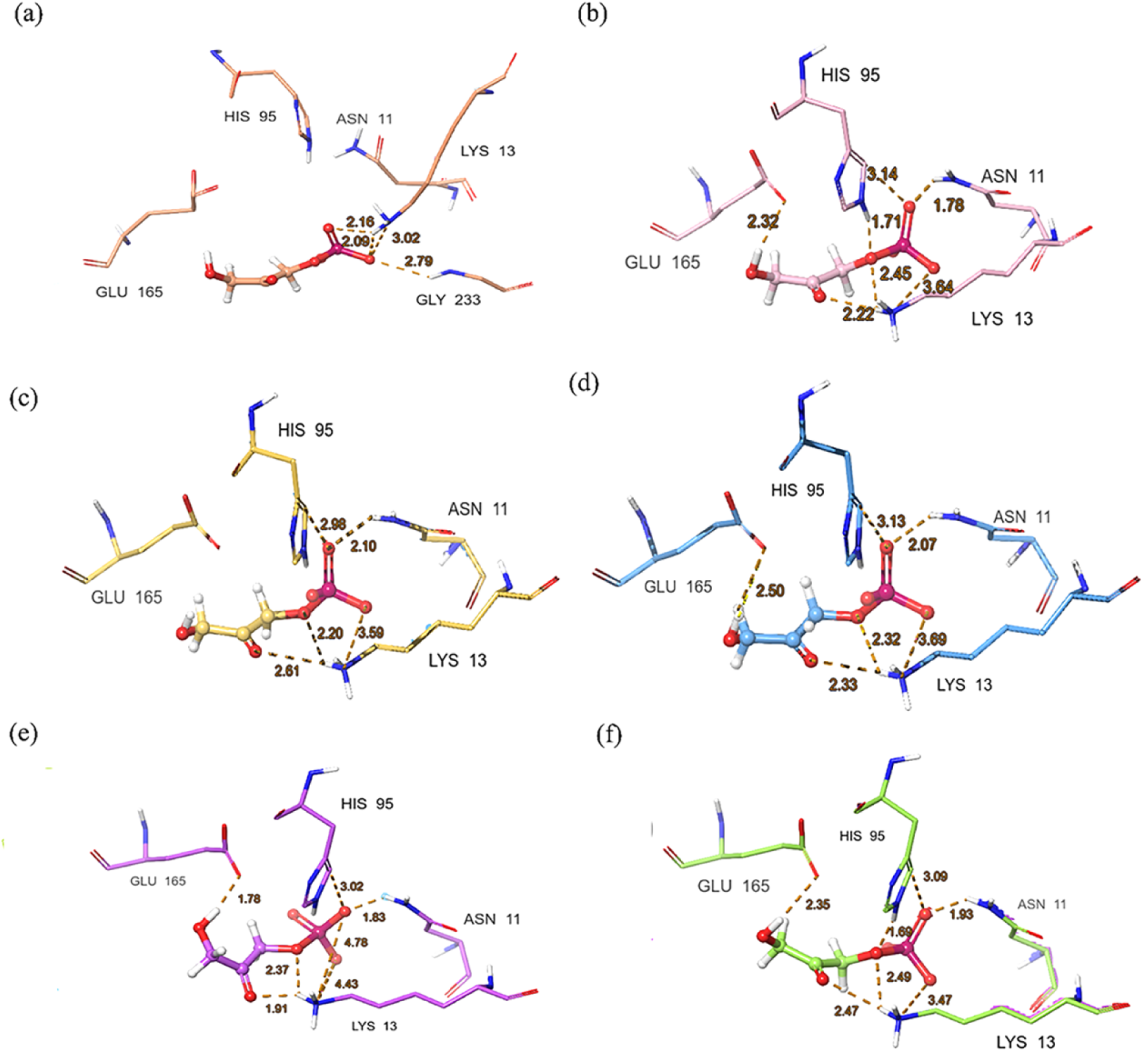
Key residue interactions and distances (in dashed lines, in Å) between DHAP and *Gg*TIM after Prime MM-GBSA calculations for conformers m1a, m1b, m2a, m2b, m3a, and m3b (a–f).

### Molecular Dynamic Simulations and Binding Free Energy Calculations

As the conformers were generated for the HR region from the intact protein structure, the restraints used in the MD simulations should be adjusted to prevent the disintegration of the truncated structure during the simulations. First, we tested three different cases using the truncated *Gg*TIM with DHAP docked to determine the harmonic restraints required (Figure S4a–c). In these cases, the truncated structure was partitioned into zones. The inner zone, comprising the catalytic site and loop 6 was unrestrained, and various harmonic restraints were employed at the other zones. For each case, 2 × 100 ns long MD simulations were conducted (Table S1), and the results were analyzed to assess ligand-protein contacts and secondary structure stability. Three cases were tested by comparing the findings with those for the dynamics of unrestrained intact *Gg*TIM dimer complexed with DHAP, taken as the base case.

Simulations of the apo *Gg*TIM-DHAP complex, which is considered the base case, showed that the complex preserved the important atom–atom interactions from the docking poses and that these interactions were maintained throughout the simulation (Figure S4d). In the docking pose displayed in Figure S3b, hydrogen bonds were observed for Asn11, Lys13, and His95 in subunit A. In addition, a hydrogen bond was formed with Ser211 in subunit B. Ligand interactions shown in the middle panel of Figure S4 indicate that DHAP maintained key interactions in the binding site throughout the simulation and established a direct hydrogen bond between the carboxylic group of Glu165 and the C=O group of DHAP. Asn11 and Lys13 formed hydrogen bonds with the phosphate group of DHAP, while His95 provided additional stabilization by interacting with the carbonyl group of DHAP. Ser211 reinforced stability in the binding site by establishing a hydrogen bond with the hydroxyl group of DHAP in the B chain.

Case 1 is similar to the local solvent boundary condition,^70^ and the truncated region was divided into three zones (Figure S4a). No force was applied to residues within the inner zone with a 15 Å radius. A harmonic restraint of 10 kcal/mol·Å^2^ was applied to residues within the 15–20 Å zone, while residues beyond 20 Å were subjected to a restraint of 50 kcal/mol·Å^2^. Considering the sharp transition from 10 to 50 kcal/mol·Å^2^, two other cases were also investigated, each dividing the truncated structure into four zones. In Case 2, the unrestrained zone was reduced to a radius of 10 Å, while applying 25 kcal/mol·Å^2^ to the 10–15 Å range, 35 kcal/mol·Å^2^ to the 15–20 Å range, and 50 kcal/mol·Å^2^ to residues beyond 20 Å (Figure S4b). In Case 3, the inner unrestrained zone had a 15 Å radius, and harmonic restraints of 10 kcal/mol·Å^2^ to the 15–20 Å range, 25 kcal/mol·Å^2^ to the 20–25 Å range, and 50 kcal/mol·Å^2^ to residues beyond 25 Å were applied (Figure S4c).

Then, we analyzed the ligand-residue interactions and the secondary structures in each case. Key residues Asn11, Lys13, His95, Glu165, and Ser211 maintained their interactions with DHAP in all three cases for all replicas, as compared to the base case, except for Case 1, where His95 did not interact with the ligand (Figure S4a) in both replicas. The percentage occurrences of these interactions are given in Figure S5a. Regarding the secondary structures, the α helix 4 (residues 95–99) accommodating His95 was not stable in both replicas of Case 1 and one replica of Case 2; and the same α helix was too stiff when compared to the unrestrained intact dimer dynamics in Case 2 (Figure S4b). Moreover, the α helix 8 (residues 156–158) was disrupted in both replicas of Case 2. The secondary structures in Case 3 were maintained when compared to the base case (Figure S4c). Figure S5b shows the average secondary structure content based on the residue index and demonstrates that the secondary structures are sensitive to restraints. Moreover, we computed Pearson correlation coefficients between the root mean-squared fluctuation (MSF) values of the truncated systems and the intact structure, only considering the unrestrained residues (Figure S5c). The correlations were moderate to high for Case 1 (in the range of 0.51–0.64) and Case 3 (in the range of 0.61–0.82), indicating that the fluctuation profiles of the unrestrained residues resembled those of the intact structure. In contrast, Case 2 exhibited a lower correlation (in the range of 0.39–0.55). These findings supported that Case 3, in particular, maintained native-like dynamics in the key functional region. Finally, MM-GBSA binding free energy calculations from the independent 100 ns simulations yielded average binding energy values of −37.22 kcal/mol for Case 1, −39.86 kcal/mol for Case 2, and −37.16 kcal/mol for Case 3, all of which are comparable to −34.48 kcal/mol of the native dimer structure. These results demonstrated that ligand binding affinity was not compromised by restraints in this region. Therefore, 3 × 300 ns long MD simulations for the DHAP docked truncated conformers were conducted following Case 3 conditions with four zones, leaving the inner zone of radius 15 Å unrestrained. We also conducted additional 3 × 300 ns long unconstrained MD simulations for the *Gg*TIM intact dimer in complex with DHAP for comparison (Table S1).

First, the analysis of the simulations for the intact apo *Gg*TIM complexed with DHAP was carried out. The RMSD of TIM slightly decreased as the simulation progressed, which could be attributed to a reduction in the mobility of loop 6. This decrease in movement is likely due to the increasing interactions between the ligand and Glu165 on loop 6 during the later stages of the simulation, stabilizing this region (Figure 4a). Figure 4a shows that the ligand remained stable in the binding site during the simulation, with an average RMSD of approximately 1 Å. Interactions of the substrate with important residues persisted for both subunits in each replicate (Figure 4b,c). Throughout the MD simulations of replica 1, interactions with key residues such as Asn11, Lys13, and Ser211 were sustained over 65% of the simulation time in subunit A. Residues Val212, Leu230, and Ala234 formed water bridges with the ligand, possibly contributing to its stabilization on the binding site. For subunit B, interactions exceeding 65% of the simulation time were observed with Asn11, Lys13, and Glu165. Additionally, a sodium ion from the surrounding environment remained proximal to the ligand for approximately 30% of the simulation duration (Figure 4c), interacting with Glu97 during this time. Consequently, the ligand displayed slightly higher mobility in subunit B. In the other two replicas, interactions with Lys13 and Glu165 were preserved, while numerous water bridges and hydrogen bonds were observed with His95 and Gly232 in the second replica (Figure S6a), and new interactions with Lys71 and Lys174 in subunit B were also noted in the third replica (Figure S6b).

**Figure 4:**
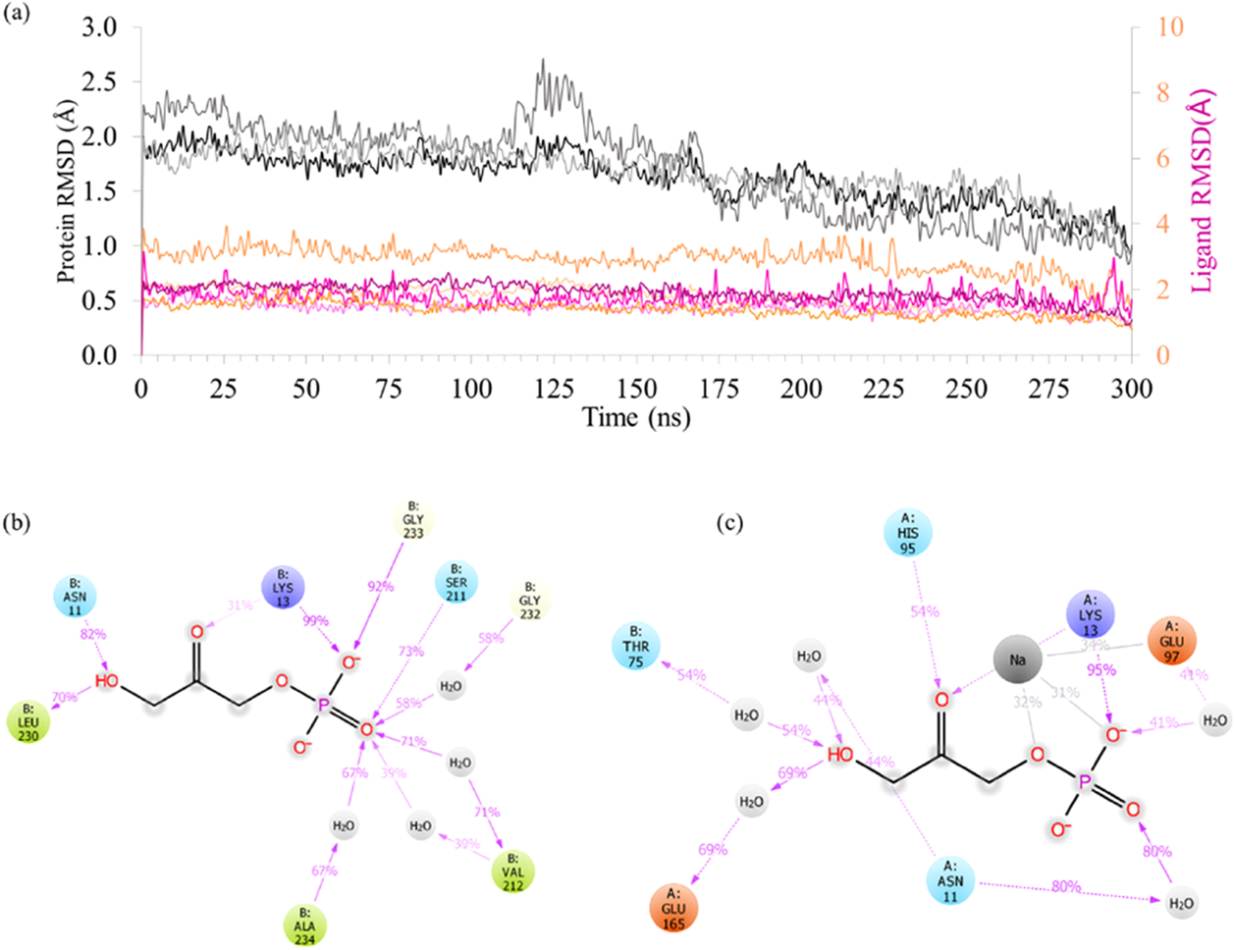
(a) The RMSD values of protein carbon alpha (black-gray) and ligands (purple tones for subunit B, and orange tones for subunit A) for all three intact *Gg*TIM replicates. (b) Interaction patterns observed in Subunit A of the intact *Gg*TIM structure. (c) Interaction patterns observed in Subunit B of the intact *Gg*TIM structure. The hydrogen bond interactions (pink arrows) observed within the binding pocket are shown for hydrophobic residues in green, polar residues in blue, and negatively charged residues in red.

In the MD trajectories of conformers obtained after ANM, truncation, and docking, both protein and ligand RMSD values generally remained below 1 Å (Figure 5a). Low RMSD of the truncated structure is expected due to the restraints. Additionally, interactions occurring in more than 30% of the simulation time for all replicates for truncated structures are illustrated in Figure S7. Here, in each mode and each replicate, DHAP interacts with key residues, corroborating the observed low RMSD values, indicating stability in the binding site (Figure 5b). However, structures derived from the slowest mode 2, i.e., m2a and m2b, exhibited the highest mobility among all, indicating a distinct level of flexibility. Moreover, MSF values for the truncated structures were analyzed, and the highest mobility was observed in residues 165–175 of the flexible loop 6 (Figure 5c). The protein–ligand interactions in the catalytic site depend on the motions of this flexible loop, which were not limited due to the truncation.

**Figure 5:**
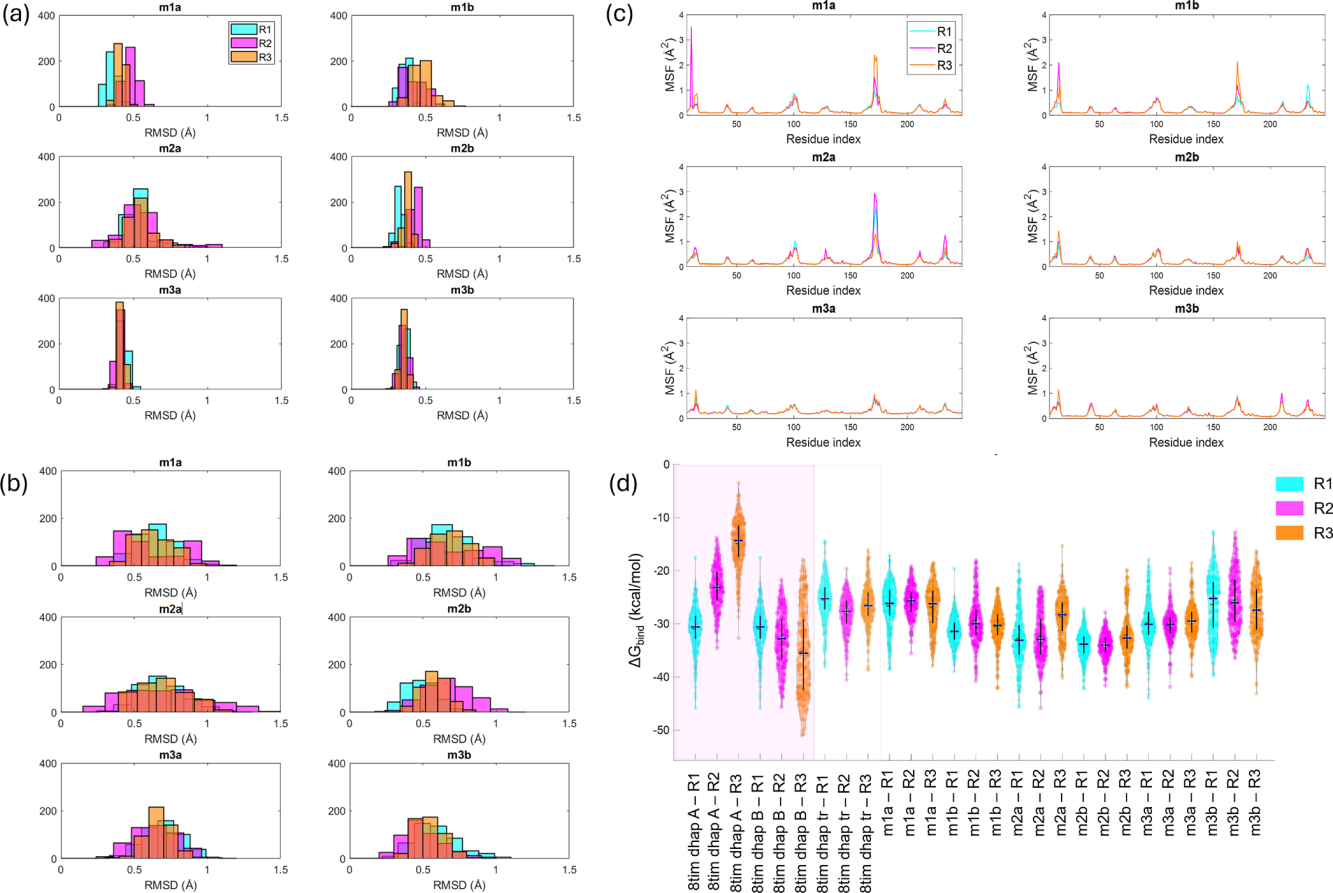
RMSD histograms of (a) the truncated *Gg*TIM conformers and (b) ligands during MD simulations. (c) MSF values for residues in subunits A and B. (c) MSF for truncated TIM structures during MD simulations. (d) ΔG_bind_ distributions calculated using MM-GBSA for all *Gg*TIM conformers generated via MCG-ANM and simulated for 300 ns. Each violin plot represents the ΔG_bind_ distribution of a specific conformer across three replicate MD simulations: R1 (cyan), R2 (magenta), and R3 (orange). The median and interquartile range are shown by black lines. The pink and gray-shaded areas indicate the nondeformed intact and truncated reference structures, respectively.

ΔG_bind_ of DHAP was evaluated across intact *Gg*TIM, truncated *Gg*TIM, and ANM-generated conformers using triplicate 300 ns MD simulations (Figure 5d). The differences between the distributions based on the replicas arise from the assignment of different initial velocities. In line with this, conducting multiple replicas is necessary to get a clear picture of the local dynamics. Previous studies investigating the effect of spherical truncation on ligand-protein interactions reported deviations up to ∼5 kcal/mol^71^ with alchemical perturbation and up to ∼1.5 kcal/mol with the MM-GBSA approach,^36^ changing according to the size of the ligand and truncation radius. In the intact structure of *Gg*TIM, the binding free energy estimations for ligands exhibited variability across chains and replicates with a few outliers, suggesting inherent fluctuations in ligand stability. Truncation led to a ΔG_bind_ of −28.08 ± 1.31 kcal/mol (run 2), which corresponds to a 7.3 kcal/mol deviation from −35.41 ± 3.64 kcal/mol (run 1) for the intact structure, where the most favorable binding free energy values from the replicates are considered (Table S3). While the harmonic restraints on the truncated structure seem to be a major source of error, this problem can be overcome by considering the ANM conformers. ANM-generated conformers restore favorable binding free energy values lost upon truncation, with ΔG_bind_ values ranging from −34.04 ± 2.27 kcal/mol to −25.49 ± 5.09 kcal/mol, closely aligning with the results for the intact structure. It is worth noting that the MM-GBSA approach neglects the entropic effects, leading to a more negative value of the binding free energy estimate for the DHAP-TIM complex, when compared to a typical binding free energy value for isomerases, ∼−5 kcal/mol.^72^ Nonetheless, the consistency across triplicate simulations reinforces the reliability of ANM-derived conformations, demonstrating that ANM-guided sampling offers a computationally efficient alternative to truncation while maintaining biologically relevant binding states and reducing the computational cost.

Comparing ANM-generated conformers, m2a and m2b modes generally showed better ΔG_bind_ values than the other conformers. In the m2b, persistent interactions with residues such as Asn11, Leu230, Gly210, and Gly209, each observed for over 90% of the simulation time across three replicates, which likely enhance the binding stability reflected in the higher ΔG_bind_ values.

Furthermore, MM-GBSA calculations were performed for all simulations, including each replicate, covering time intervals of the first 50, 100, 200 ns, and the entire 300 ns duration (Figure S8 and Table S3). This comprehensive analysis resulted in consistent ΔG_bind_ across all time intervals. The uniformity in ΔG_bind_ calculations throughout various time intervals underscores the reliability of the docking poses. Additionally, based on this uniformity, the initial 100 ns were deemed sufficient for accurately estimating the restrained dynamics involving a flexible loop over a catalytic site.

By leveraging insights from ANM, the truncated structures seemed to provide various conformations of the catalytic site, comparable to the intact structure dynamics, enabling computationally efficient exploration of conformational space without compromising structural integrity.

### The Interface Region of *Pf*TIM

#### Conformer Generation Using Mixed Coarse-Graining

The dimer interface of *Pf*TIM is another ligand binding site investigated, where 3-phosphoglyceric acid (3PG) interacts with residues Asn65, Arg98, and Lys112, all conserved in other species, as well as the nonconserved Lys68, Phe102, and His103.^73^ In the MCG ANM calculations, we considered the apo *Pf*TIM (PDB ID: 1VGA). A spherical region with a 30 Å radius centering the binding site of 3PG at the interface was modeled at atomic resolution, while the remaining protein surface was represented at LR (Figure 6a). The dynamic behavior of the structure predicted by the MCG model was evaluated against experimental B-factors (Figure S9), resulting in a correlation coefficient of 0.72, which indicates a good level of agreement. The collective motions determined by ANM analysis were observed as twisting and two different bending-type movements for the first three modes, respectively (Figure 6b–d), as in the former case study on *Gg*TIM. Figure 6e shows the motions of loop 3, where the ligand interacts at the dimer interface, in different directions in the first three modes. Mode 1 corresponds to a lateral shift in loop 3; mode 2 involves a slight inward bending; and mode 3 shows a stronger inward bending motion, creating the effects of expanding or shrinking the ligand binding site.

**Figure 6:**
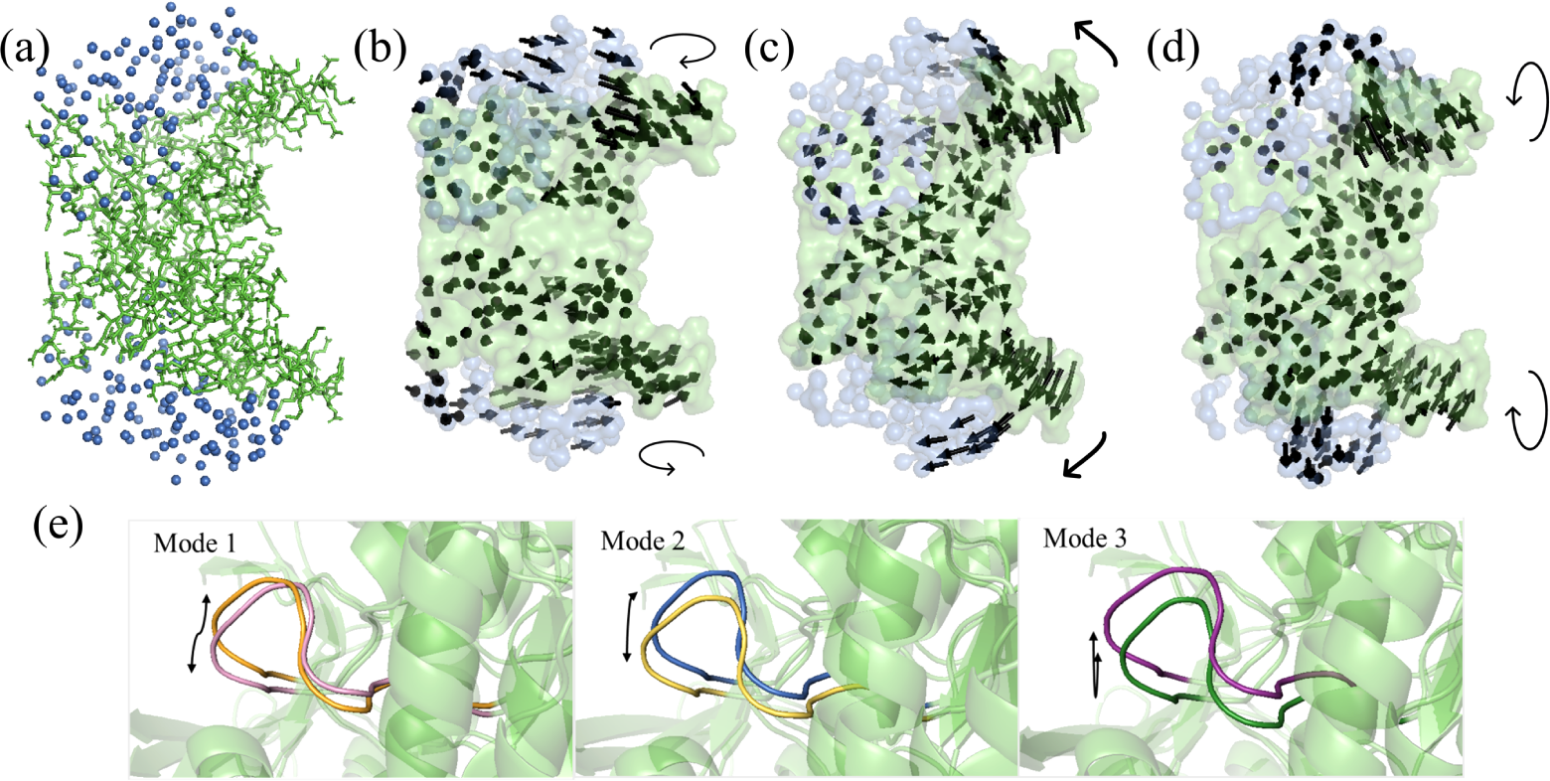
(a) MCG structure of *Pf*TIM, with HR (green sticks) and LR (blue spheres) regions. Displacement vectors are displayed for (b) Mode 1, (c) Mode 2, and (d) Mode 3. For clarity, the motions are exaggerated and only shown for Cα atoms using arrows. (e) Motions of loop 3 at the dimer interface in different directions in the first three modes are shown.

36 conformers were produced for three modes by applying six different deformations (1.0, 1.5, 2.0, 2.5, 3.0, 4.0 Å). HR regions from the conformers were truncated and subjected to energy minimization. Figure 7a,b shows the deformations of loop 3 in the positive and negative directions of the slowest mode, respectively, to illustrate the extent of its motions at the dimer interface. Figure 7c displays the energy values of the minimized conformers, compared with the minimum truncated *Pf*TIM structure, in the form of energy difference. The energy values are either lower or slightly higher than the minimum structure at deformations of 1.0 Å and 1.5 Å, where the structural integrity is preserved. For the conformers with deformations of 2 Å and above, the energy values are higher than those of the minimum structure. Unlike *Gg*TIM (Figure 2c), high structural deviations in *Pf*TIM were retained after minimization, as it is noted with the postminimization RMSD (rmsd_min) values increasing with deformation RMSD values from 1.0 to 4.0 Å. For a few numbers of mode shapes, a high structural deformation resulted in energetically favorable conformers. The comparison between the two HR regions (i.e., the catalytic site of *Gg*TIM and the dimer interface of *Pf*TIM) revealed distinct behaviors, underscoring the need for system-specific calibration when determining the extent and direction of deformations. Consequently, the conformers generated with deformations of 1.0 and 1.5 Å were determined as suitable for molecular docking studies.

**Figure 7:**
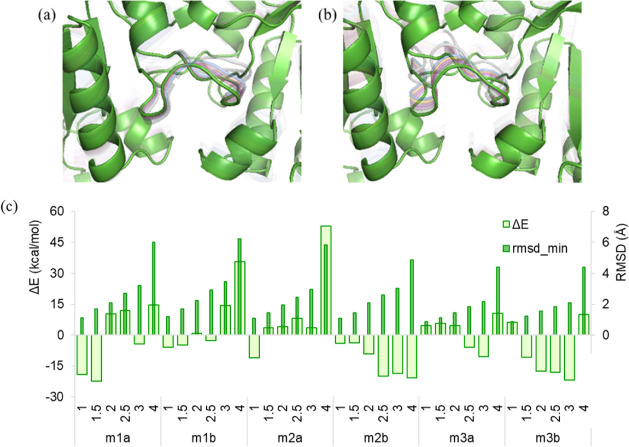
(a) Positive and (b) negative direction displacements of the dimer interface with loop 3 in Mode 1 with deformations from 1 Å to 4 Å. (c) Energy differences between the energetically minimized conformer and the minimum structure (ΔE), and postminimization RMSD values (rmsd_min) for *Pf*TIM are plotted against the deformation RMSD values (1.0–4.0 Å) for each mode (m1a-m3b), given in the *x*-axis.

### Molecular Docking Studies

The docking procedure was validated with 3PG using the holo *Pf*TIM structure (PDB ID: 2VFI). The crystal structure contains two 3PG molecules at the dimer interface, where ligand binding sites are symmetrical. In the crystal structure, the ligand establishes hydrogen bonds and electrostatic interactions with Asn65, Lys68, Phe102, and Glu104 in subunit A, while a similar pattern of interactions is symmetrically observed in subunit B (Figure S10a,b). The crystal pose of 3PG was generated in both subunits with an RMSD value of nearly 0 Å. Prime MM-GBSA calculations for the crystal pose reported −16.22 kcal/mol for subunit A and −15.77 kcal/mol for subunit B (Table 2). The interactions observed in the crystal structure are preserved, except for the extra Lys68 salt bridge in subunit B due to the flexibility of the residues in the Prime calculations (Figure S10c,d).

**Table 2:** Prime MM-GBSA Values for the Ligands Docked to the *Pf*TIM Dimer Interface^a^ ^b^

Structure	Ligand	RMSD before minimization (Å)	RMSD after minimization (Å)	Prime MM-GBSA (kcal/mol)
Holo *Pf*TIM (subunit A)	3PG	-	0.0	–16.22
Holo *Pf*TIM (subunit B)	3PG	-	0.0	–15.77
Apo *Pf*TIM (subunit A)	3PG	-	0.8	–5.90
Apo *Pf*TIM (subunit B)	3PG	-	0.7	–6.45
Apo *Pf*TIM (truncated)	3PG	-	1.0	–5.67
*Pf*TIM_m1a	3PG	1	1.9	–8.25
*Pf*TIM_m1b	3PG	1	1.0	–16.03
*Pf*TIM_m2a	3PG	1	1.9	–21.28
*Pf*TIM_m2b	3PG	1	1.3	–13.12
*Pf*TIM_m3a	3PG	1	1.4	–21.01
*Pf*TIM_m3b	3PG	1	1.4	–17.37
*Pf*TIM_m1a	3PG	1.5	1.2	–15.68
*Pf*TIM_m1b	3PG	1.5	1.0	–30.43
*Pf*TIM_m2a	3PG	1.5	1.2	+10.05
*Pf*TIM_m2b	3PG	1.5	1.4	–3.81
*Pf*TIM_m3a	3PG	1.5	1.3	–19.21
*Pf*TIM_m3b	3PG	1.5	1.2	–7.34

a The root mean-squared deviations (RMSD) of the conformers before and after energy minimization are also listed

b The reference structure is apo *Pf*TIM (truncated).

The ligand was then docked to the apo *Pf*TIM (PDB ID: 1VGA) dimer interface. In the docking results for the apo *Pf*TIM structure, the ligand largely preserved the binding motifs of the crystal holo-structure by establishing hydrogen bonds with Asn65, Glu104, and Phe102 in subunit A (Figure S10c). Similar hydrogen bonding and/or salt bridges were observed with Lys68, Arg98, and Asp103 in subunit B (Figure S10d). However, in the apo structure, docking 3PG into subunit A showed a reduced XP score (Table S2, −2.15 kcal/mol) compared to subunit B (Table S2, −6.02 kcal/mol), despite comparable Prime MM-GBSA results (−5.90 kcal/mol for subunit A and −6.45 kcal/mol for subunit B), and similar poses in both chains. This discrepancy seems to arise due to steric hindrance from the ligand already docked in subunit B, which interfered with the subsequent docking into subunit A. When the ligand was docked into subunit A in the absence of 3PG in subunit B, the XP score improved to −6.06 kcal/mol, aligning with subunit B results of −6.02 kcal/mol. Prime MM-GBSA value also remained consistent (−6.45 kcal/mol). Furthermore, removing the ligand from subunit B after docking and performing a flexible Prime calculation for subunit A improved the Prime MM-GBSA value to −16.00 kcal/mol, demonstrating the impact of steric effects in the apo structure. Then, docking calculations were performed for the truncated apo *Pf*TIM structure and the derived conformers, with the ligand was exclusively docked into subunit B. All subsequent calculations, including Prime MM-GBSA analyses, were conducted based on this setup, maintaining the focus on the interactions within subunit B. The binding pattern of the ligand was noted to be preserved in the truncated apo *Pf*TIM structure (Figure S10g).

Prime MM-GBSA values for the apo and truncated structure were lower than the holo-structure, likely due to conformational differences in the binding pocket, such as the altered positioning of Lys68. Docking calculations were performed on conformers generated from apo *Pf*TIM with RMSD values of 1.0 and 1.5 Å. Docking on apo *Pf*TIM conformers with 1.5 Å deformation yielded a positive Prime MM-GBSA energy for the conformer *Pf*TIM_m2a (+10.05 kcal/mol), likely due to interface deformation misaligning the ligand in the docking grid. Therefore, docking analyses were continued with conformers with a deformation RMSD of 1.0 Å.

Conformers with a deformation RMSD of 1.0 Å showed various conformational flexibility compared to the crystal pose. The ANM conformers preserved key interactions of 3PG in the binding pocket of the crystal structure while offering a wider range of flexibility. Although the XP scores were relatively low due to rigid docking (Table S2), the Prime MM-GBSA values reached similar values to the holo-structure since the residues were kept flexible (Table 2). The crystal structure interactions (Figure S10a,b) reveal that the phosphate group interacts with Asp65 and Arg98, while the carboxyl group engages with His103 and/or Lys68 in both subunits. In addition, the hydroxyl group interacts with Lys68 in subunit B. In the ANM-generated conformers, Asp65 and Arg98 remain key interacting residues with the phosphate group, and the hydroxyl group interacts with Phe102 across all modes. Except for *Pf*TIM_m2a and *Pf*TIM_m3b, the carboxyl group retains its interaction with Lys68 in the conformers (Figure 8). Prime MM-GBSA results for *Pf*TIM_m1a and *Pf*TIM_m1b were calculated as −8.25 kcal/mol and −16.03 kcal/mol. *Pf*TIM_m1b exhibited a docking pose with improved binding affinity, interacting with Asn64, Lys67, and Arg97 through hydrogen bonds and salt bridges (Figure 8a,b). The Prime MM-GBSA results reached −21.28 kcal/mol and −13.12 kcal/mol for the second mode. *Pf*TIM_m2a stood out as the most energetically favorable structure with the lowest Prime MM-GBSA value due to the strong interactions of the ligand with Phe101, Lys67, and Asn64 (Figure 8c,d). Prime MM-GBSA results in *Pf*TIM_m3a and *Pf*TIM_m3b conformers were calculated as −21.01 kcal/mol and −17.37 kcal/mol, respectively. In both modes, the ligand established strong interactions with Lys67, Phe101, and Asn64, and energetically favorable binding poses were obtained (Figure 8e,f).

**Figure 8:**
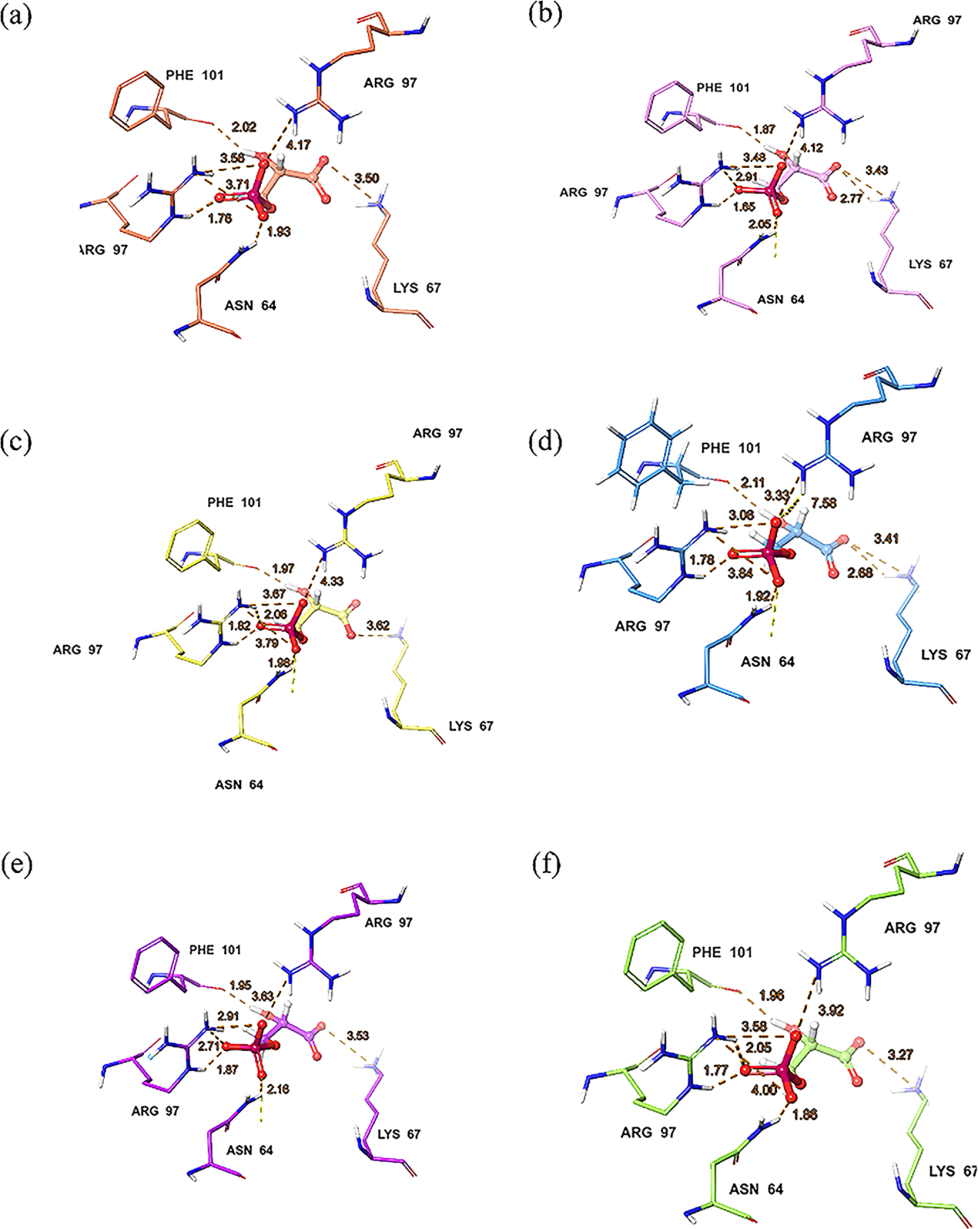
Key residue interactions and distances (Å, dashed lines) of Prime MM-GBSA poses of 3PG at the *Pf*TIM dimer interface for the ANM conformers (a) *Pf*TIM_m1a, (b) *Pf*TIM_m1b, (c) *Pf*TIM_m2a, (d) *Pf*TIM_m2b, (e) *Pf*TIM_m3a, and (f) *Pf*TIM_m3b.

Prime MM-GBSA values showed that the binding poses of 3PG in the conformers were energetically more favorable when compared to the docking results to the apo-structure, underlining that ANM successfully generated different conformations of the binding site from the apo form. Compared to validation docking to the crystal holo-structure, the ligand-protein interactions in the conformers were diversified, especially from the carboxylic end of 3PG that maintained the key interactions. RMSD values after minimization, reflecting the deviation of the predicted binding pose from the reference structure, also remained between 1.0 and 2.0 Å, indicating that the overall stability of the structures was maintained.

### Molecular Dynamics Simulations and Binding Energy Calculations

First, MD simulations were performed on the intact apo-structure with ligands docked into subunits A and B as the reference case. The RMSD profile based on α carbon atoms, excluding the highly mobile residues (residues 3–4, 171–173, and 194–196), showed that the enzyme reached an RMSD of ∼2 Å in all replicas. The ligands in subunits A and B remained relatively stable during the simulations, especially up to 150 ns (Figure 9a). In some replicas, the ligand RMSD increased up to 3.0 Å without leaving the binding pocket. Simulations for the apo *Pf*TIM structure showed that the ligand maintained the interactions in its binding pose and continued to establish hydrogen bonds and electrostatic interactions with key residues such as Asn65, Lys68, and Glu104 (Figure 9b,c). In addition, Asp108 and Arg98 in subunit B established hydrogen bonds or water bridges with 3PG. These interactions in the apo structure were similar to the crystal interactions of the holo-structure assessing the docking simulations. Similar bindings continued with Asn65, Lys68, and Glu104 in subunits A and B in the second and third replicas (Figure S11).

**Figure 9:**
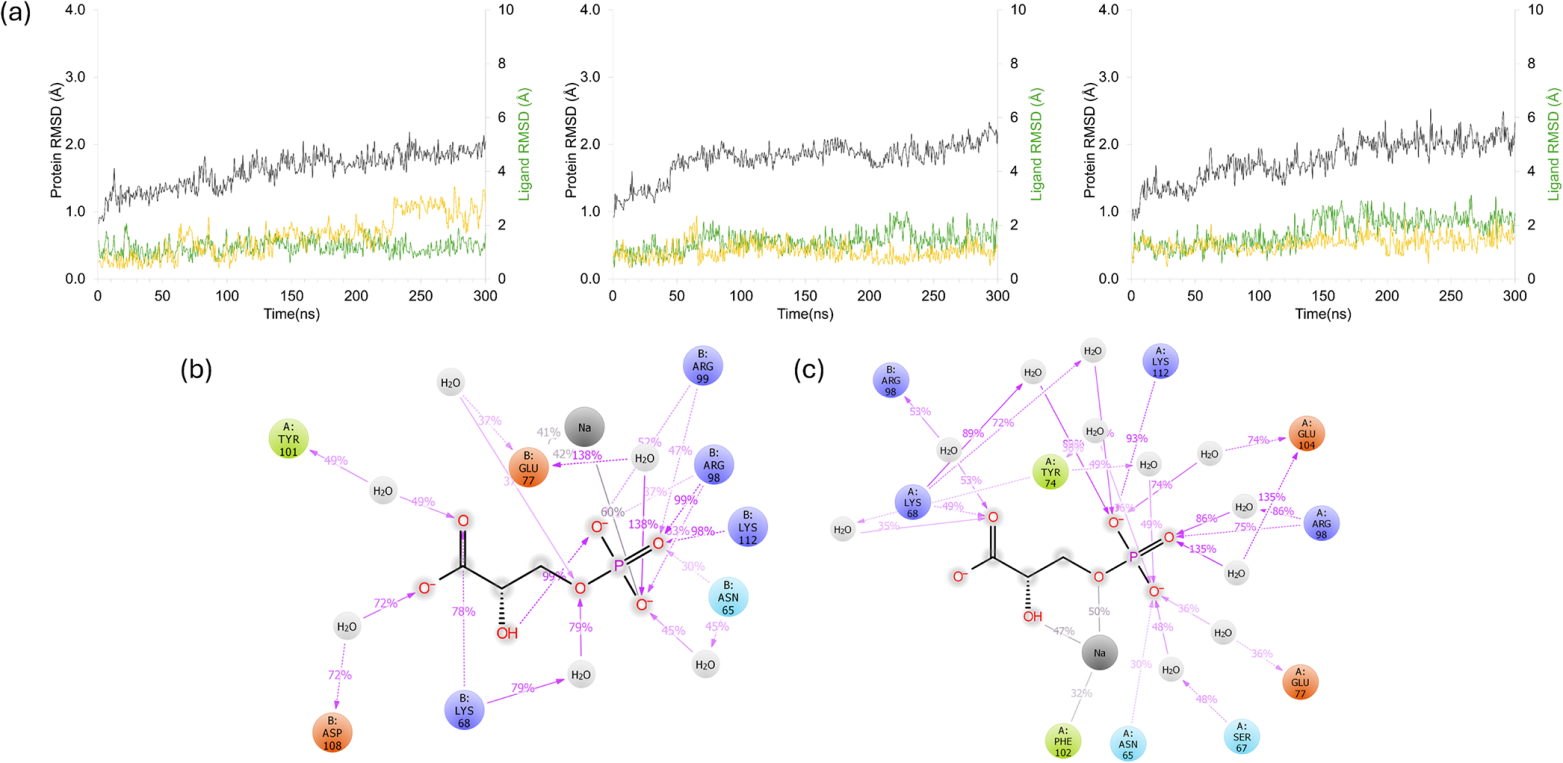
(a) The RMSD values of the intact *Pf*TIM (gray tones) and ligands (yellow tones for 3PG in subunit A, green tones for 3PG in subunit B), shown for the three replicates from R1 to R3. 2D interaction maps in (b) subunit A and (c) subunit B. The coloring in (b) and (c) is as in Figure 4.

MD simulations were performed with the ANM conformers using the set of harmonic restraints applied in four zones. The RMSD values (Figure 10a) indicate that the truncated conformers retain their structural integrity, and the ligands remain in the binding pockets. In the independent simulations for the truncated *Pf*TIM conformers, docked ligands maintained hydrogen bonding and electrostatic interactions with the key residues (Figure S12). Despite variations in interaction frequencies and additional contacts across modes, consistent key interactions—Asn65 and Arg98 in *Pf*TIM_m1a/b, Lys67 and Tyr102 in *Pf*TIM_m2a/b, and Asp108 and Glu104 in *Pf*TIM_m3a—were maintained, reflecting expected binding patterns and highlighting the binding region’s flexibility. These findings indicate that the generated conformers are plausible.

**Figure 10:**
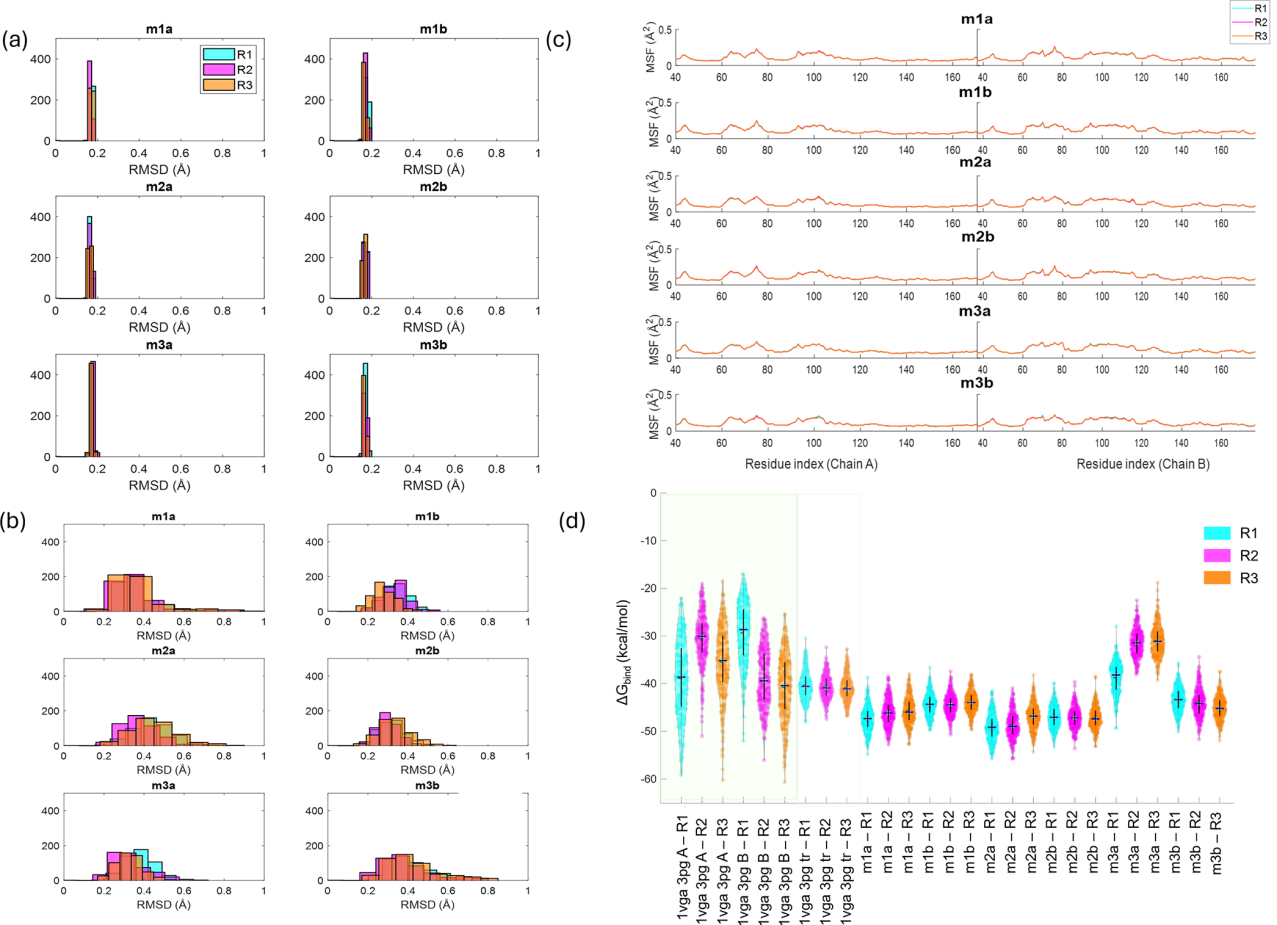
RMSD histograms of (a) the truncated *Pf*TIM conformers and (b) ligands during MD simulations. (c) MSF values for residues in subunits A and B. (d) ΔG_bind_ distributions calculated using MM-GBSA for all *Pf*TIM conformers generated via MCG ANM and simulated for 300 ns. Each violin plot represents the ΔG_bind_ distribution of a specific conformer across three replicate MD simulations: R1 (cyan), R2 (magenta), and R3 (orange). The median and interquartile range are shown by black lines. The green- and gray-shaded areas indicate the nondeformed intact and truncated reference structures, respectively.

ΔG_bind_ of 3PG at the *Pf*TIM dimer interface was evaluated using 300 ns MM-GBSA calculations across the intact structure, the truncated interface region, and ANM-generated conformers (Figure 10d and Table S3). In the intact system, ligand binding in chain A exhibited greater variability across replicates, indicating a more flexible binding environment, whereas chain B showed lower variance. The binding free energy decreased by 1.54 kcal/mol from −38.95 ± 7.85 (intact, run 1) to −40.49 ± 2.74 (truncated, run 3) upon truncation, indicating enhanced binding stability despite the removal of structural elements at the interface.

ANM-generated conformers displayed binding free energy values comparable to the intact structure (between −47.23 ± 2.61 and −31.37 ± 2.91 kcal/mol), with m2a and m2b showing particularly favorable ΔG_bind_ values. The lower variance across replicates in ANM conformers suggests that these structures capture stable, functionally relevant binding states, which can be lost upon truncation. This highlights the ability of ANM sampling to restore ligand-binding stability while reducing computational cost, reinforcing its value as a conformational sampling strategy in dynamic protein–ligand interactions.

Thermal MM-GBSA calculations were also performed for the periods of 50, 100, 200, and 300 ns to determine the optimum simulation length for the estimation of binding free energy values of the docked compounds (Figure S13). The results showed consistent ΔG_bind_ values for both subunits of the intact *Pf*TIM and the truncated conformers, indicating that binding energies remained consistent across these periods. Accordingly, a simulation time of 100 ns can provide reliable results for this truncated system, focusing only on the binding site.

### Docking 3PG to the *Gg*TIM Dimer Interface

The computational approach presented in this study succeeded in reproducing the native ligand-protein interactions at the binding sites on the conformers obtained from the apo structures of TIM. The catalytic site accommodates highly conserved amino acids across species, while the dimer interface involves nonconserved residues, such as Lys68, Phe102, and His103.^73^ This raised the question of whether 3PG, the native ligand of *PfI*TIM holo-structure dimer interface, could bind the dimer interface of *Gg*TIM, and if so, similar interaction patterns could be observed.

Molecular docking studies showed that 3PG could be docked to the dimer interface of apo *Gg*TIM but with a pattern different than that of *Pf*TIM (Figure S14a,b) due to the steric hindrance by the side chain of Glu104, as seen in Figure 11. In subunit A, the ligand changed its orientation in the binding pose such that Lys68 interacted with the phosphate group of 3PG while it was expected to interact with its −COO^–^ group. In subunit B, 3PG bound another region, away from its binding site. In line with this, XP scores in the intact *Gg*TIM structure were low (Table S2, subunit A: −2.93 kcal/mol, subunit B: −3.07 kcal/mol), as well as the Prime MM-GBSA values (subunit A: −6.12 kcal/mol, subunit B: −7.76 kcal/mol) (Table 3). Then, 3PG was docked to the truncated *Gg*TIM. Although a high Prime MM-GBSA value was obtained (−23.41 kcal/mol), the orientation of 3PG was incorrect (Figure S14c).

**Figure 11:**
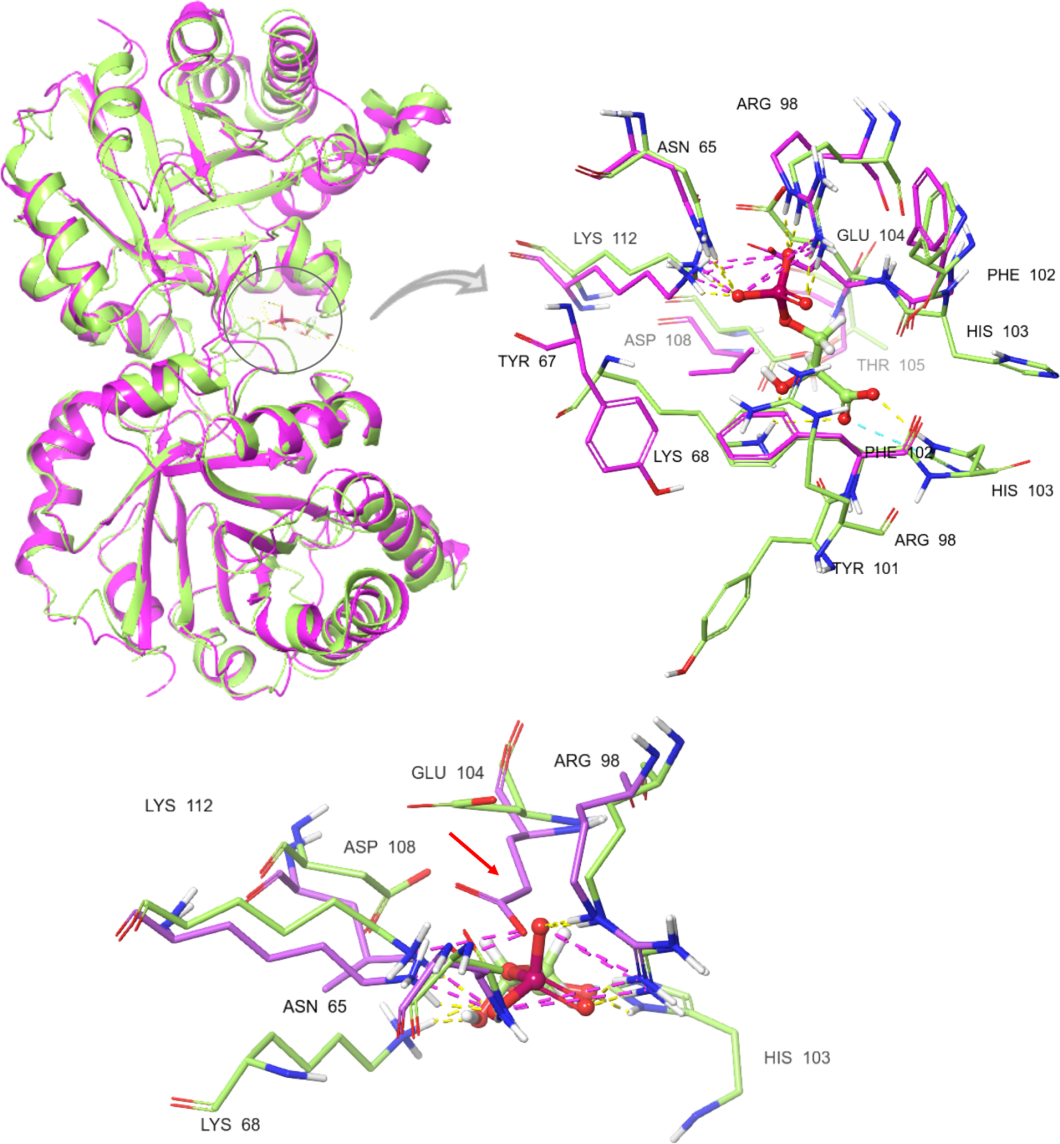
*Pf*TIM (green, PDB ID: 2VFI) and *Gg*TIM (pink, PDB ID: 8TIM) superimposed. The encircled area is enlarged to show the differences in residue orientations at the ligand binding sites from different perspectives. The red arrow indicates the most pronounced difference (Glu104) between the species.

**Table 3:** Prime MM-GBSA Values for the Ligands Docked to the *Gg*TIM Dimer Interface^a^ ^b^

Structure	Ligand	Deformation RMSD (Å)	RMSD after minimization (Å)	Prime MM-GBSA (kcal/mol)
Holo *Pf*TIM (subunit A)	3PG	-	0.1	–16.22
Holo *Pf*TIM (subunit B)	3PG	-	0.1	–15.77
Apo *Gg*TIM (subunit A)	3PG	-	0.2	–6.12
Apo *Gg*TIM (subunit B)	3PG	-	0.2	–7.76
Apo *Gg*TIM (truncated)	3PG	-	0.1	–23.41
Apo *Gg*TIM_m1a	3PG	1.0	1.2	–6.33
Apo *Gg*TIM_m1b	3PG	1.0	1.2	–40.77
Apo *Gg*TIM_m2a	3PG	1.0	1.5	–25.80
Apo *Gg*TIM_m2b	3PG	1.0	1.0	–20.97
Apo *Gg*TIM_m3a	3PG	1.0	1.4	–19.07
Apo *Gg*TIM_m3b	3PG	1.0	1.5	+27.43

a The root mean-squared deviations (RMSD) of the conformers before and after energy minimization are also listed.

b The reference structure is apo *Gg*TIM (truncated).

Taking the interface region at HR, apo *Gg*TIM conformers were generated using MCG ANM, with a deformation factor of 1.0 Å from the minimum structure, similar to previous case studies. The XP scores (Table S2) were relatively low compared to the holo *Pf*TIM, while the Prime MM-GBSA values were improved, as shown in Table 3. The ligand binding pose displayed a similar orientation as in *Pf*TIM with favorable interactions for *Gg*TIM_m2a, *Gg*TIM_m2b and *Gg*TIM_m3a (Figure S14d–i). In contrast, for *Gg*TIM_m1a and *Gg*TIM_m3b, the ligand docked to a different site, and for *Gg*TIM_m1b, the orientation of the ligand was different. This highlights that ANM was able to provide a geometrically suitable binding site for the ligand. Nonetheless, the docking results for *Pf*TIM and *Gg*TIM dimer interfaces reveal the critical structural differences between the two species influencing 3PG binding.

### Computational Efficiency of the MCG ANM Coupled with Truncation

The current method is developed to provide plausible conformers for ensemble docking and overcome the computational limitations faced in the ligand binding free energy calculations for large protein complexes, such as the 2.5 MDa bacterial ribosome, by reducing system size with truncation. In the current practice, the conformational flexibility of the systems, including the bacterial ribosome, can be explored with computationally efficient CG and all-atom/CG models, where conformer generation is on the order of minutes for ENM-based techniques,^63,64^ including the MCG ANM. The MCG ANM successfully captured the conformational changes of TIM at mixed resolution, together with loop 6 dynamics observed in the crystal structures and classical NMA.^50^ On the other hand, other hybrid resolution techniques, such as local replica exchange MD,^13,74^ accelerated MD,^14,75^ MARTINI-based methods,^21,76,77^ and PRIMO model,^20^ also offer high computational efficiency in exploring conformational dynamics. Local replica exchange MD simulations can focus on a small region of interest while maintaining the full structure. Similarly, accelerated MD can assign multiple boosting potentials to numerous regions of interest, but keeps the full structure.^78^ MARTINI-based coupling schemes typically assign distinct resolutions to different components of the system, for example, proteins at all-atom resolution and membranes or solvents at CG resolution, without mixing resolutions within the same chain. This can set a limitation when the truncated structure may need to involve multiple chains, as in the TIM dimer interface presented as the second case study. PRIMO supports mixed-resolution representations within a single biomolecule, while additional tuning of nonbonded interactions is required to optimize inter-region compatibility.^20^

The AI-assisted AlphaFold2 (AF2), originally designed to predict protein structures, was shown to generate native-like conformations of proteins that can be exploited in drug design.^79^ Here, by manipulating the setting parameters in the AF2 server and its accelerated version ColabFold,^57^ we explored the capability of AF2, as a comparative and efficient tool to assess conformational diversity of TIM, focusing on the dimer interface and the flexible loop 6 that was shown to adopt intermediate and open conformations according to MCG ANM calculations (Figure S15a). AF2, which allows the prediction of dimer structures, produced 12 native-like conformers with high predicted local distance difference test (pLDDT) and predicted template modeling (pTM) scores (Table S4). In modeling TIM dimers based on a template, loop 6 was likely to assume a closed conformation similar to the holo structure, whereas AF2 tended to produce more open conformations without a template (Figure S15b). In the monomer generation, the conformations of loop 6 clustered around the closed state, regardless of the presence of a template. These observations support previous findings that AF tends to favor compact, holo-like states (∼70% of predictions), even in template-free setups^80,81^ limiting conformational selection in drug discovery applications. On the other hand, ColabFold, which currently allows the modeling of only monomeric structures, enabled testing the effect of multiple sequence alignment (MSA) depth on protein structure prediction. With a single-sequence input strategy without providing MSA, predicted models exhibited unrealistic folding, with significant distortions even in well-structured regions such as the TIM barrel, reflected by a very low confidence level (pLDDT < 50). When MSAs were automatically generated using MMSeqs2, structurally reliable models were produced with high pLDDT scores, but with limited conformational diversity in the loop 6 region, despite introducing different seeds to encourage stochastic variation (cf1 in Table S4). Then, the depth of the MSA was modulated by setting the max_msa_clusters and max_extra_msa parameters to 16 and 32, respectively, reducing the evolutionary information available to the model. This resulted in well-folded structures (pLDDT > 90) exhibiting greater variability in the conformation of loop 6 (cf2-cf4 in Table S4, Figure S15c). Finally, a higher MSA depth setup was explored (max_msa_clusters = 64, max_extra_msa = 128) that produced plausible models but with reduced structural diversity, similar to the full-MSA condition (cf5-cf6 in Table S4, Figure S15c). AF2 and ColabFold successfully predicted different conformers for loop 6 in the order of minutes. The models with open-loop 6 conformations with high confidence scores are suitable for molecular docking. However, even if AF2 could predict the quaternary structure correctly, it could not produce different conformers of the dimer interface (Figure S15d). It should also be noted that the maximum allowed sequence length per structure (currently 4 × 10^3^ for multimer, 2.5 × 10^3^ for monomer) can limit the generation of very large protein complexes, such as the bacterial ribosome. Another current limitation of AF2 is that it cannot predict the metals, cofactors, ions, and water molecules that can be crucial in drug design. MCG ANM can include these nonprotein components as nodes into the elastic network, when desired.

The generated conformers are used in ligand docking. However, scoring functions may be unreliable for ranking the docked compounds and remain insufficient to reveal the local dynamics of the ligands in the binding site. MD simulations coupled with free energy perturbation, thermodynamic integration, or MM-GBSA/PBSA methods can provide such detailed analysis. Thus, the proposed method offers an alternative to all-atom MD simulations of intact structures coupled with binding energy calculations, which represent the benchmark for structure-based binding energy analysis for ligand ranking. In Table 4, the computational efficiency of the method is shown by comparing simulation rates in Desmond for the truncated and intact structures of TIM from this study and the bacterial ribosome from other studies. Desmond’s GPU implementation runs each trajectory on a single GPU, meaning that reducing the atom count directly improves simulation rates. Here, the CPUs to initiate the simulations may change from one system to the other, but not affect the simulation rates. For the TIM structures, truncation reduced the simulation times slightly. Computational efficiency was more pronounced when the intact and truncated bacterial ribosomes were compared, where simulations were performed using GPUs with similar performance. In the Desmond/GPU performance report published by Shaw et al.,^82^ benchmark results show that large systems like the ribosome, containing over 2 million atoms, require high GPU memory and substantial computing time, even under optimized simulation settings. In such large systems, the MCG-based truncation can enable multiple simulations for ligand ranking purposes that would otherwise be infeasible on standard GPU hardware.

**Table 4:** Simulation Rates of Intact and Truncated Structures in Desmond

System	Total number of atoms	GPU	Time step	Simulation time (ns/day)
*Gg*TIM intact structure	4.2 × 10^4^	NVIDIA A100 GPU	2 fs	95.2
*Gg*TIM truncated structure	3.4 × 10^4^	NVIDIA A100 GPU	2 fs	117.5
*Pf*TIM intact structure	4.1 × 10^4^	NVIDIA A100 GPU	2 fs	84.8
*Pf*TIM truncated structure	2.6 × 10^4^	NVIDIA A100 GPU	2 fs	105.6
Ribosome 70S intact structure^82,83^	2.2 × 10^6^	GeForce GTX 1080 Ti	2.5 fs	8.6
Ribosome truncated structure^49^	2.3 × 10^5^	NVIDIA Tesla V100	2 fs	26.5

## Conclusions

This study presents a computationally efficient method for calculating ligand binding free energies using truncated ANM conformers. Different binding sites of the glycolytic enzyme TIM, catalytic and dimer interface regions, were investigated using two different organisms, and . Mixed-resolution ANM conformers from the slowest three modes were created with the binding sites modeled at high resolution, and the remaining structure at low resolution. The high-resolution regions of the conformers were truncated and deformed to different extents. The truncated conformers with an RMSD value of 1.0 Å were proved to give plausible docking poses of the ligands out of different conformers with up to 2.0 Å deformation. Independent MD simulations demonstrated the stability of the ligands docked at the selected conformers. Results showed that the desired conformational diversity in the ligand binding pockets was achieved, and the conformers provided an extended conformational space to monitor the key interactions with the ligands, which could be achieved by multiple independent long simulations of the intact structures. ΔG_bind_ values were estimated for the ligands docked at the conformers using the MM-GBSA approach, while neglecting the entropic term, at different periods (50, 100, 200, and 300 ns). The results were consistent for all periods, implying that shortened simulation times, such as 100 ns, can also offer sufficient accuracy to estimate ΔG_bind_ values for the restrained truncated structure of TIM. However, it is worth noting that this may not be the case for systems with large binding sites and slow relaxation dynamics.

The MCG ANM captured the globular motions of apo *Gg*TIM during the MD simulations, with a cumulative overlap value of 0.58 averaged over the three principal components and slowest modes, where the hinge motions of loop 6 and the dimer interface are observed. Thus, the three slowest modes were useful to describe the conformational flexibility around the binding sites. However, the selection of the normal modes may differ from one protein to another, where local loop dynamics and binding site opening may be observed at the intermediate-frequency range, and a relevant mode needs to be revealed.^84^ More modes from the low-to-intermediate-frequency range can be considered in the calculations to enrich the conformational diversity, depending on the protein structure.

Furthermore, while ANM does not explicitly incorporate electrostatics or long-range interactions, studies have shown that it can still recapitulate key motions relevant to binding and catalysis,^85^ and the opening/closing motions of functional loops over the catalytic site.^41^ The local binding region from the ANM-deformed structures, when subjected to docking and short MD simulations, can undergo ligand-induced local adaptations effectively incorporating a form of induced fit at the local scale.^30^ Therefore, this approach can be readily extended to capture intermediate conformational states with ligand-induced fit effects using a cycle of MCG ANM, energy minimization, docking, and MCG ANM. For large and highly flexible systems, such as calmodulin or adenylate kinase, this localized and modular framework can offer a practical computational advantage, with potential future applications to iterative conformer–docking–refinement cycles. This method can be also beneficial for investigating the local dynamics of the residues at a binding site to monitor active site closure, loop rearrangements, intermediate conformational states, to quantify the change in the binding affinity as a result of a nucleotide/residue mutation at the binding site, or to reveal the favorable binding mode of the ligand. On the other hand, the generated mixed-resolution conformers can be subjected to backmapping to perform further all-atom conformations for conformational sampling. While backmapping is not readily available in the method, it can be achieved by completing the CG residue with the atoms displaced using the same magnitude and direction of the CG node.

While the proposed mixed coarse-grained ANM and truncation-based approach depends on the force field for binding free energy calculations, it is expected to speed up the ligand rankings after molecular docking calculations using large compound libraries. Unlike methods that apply restraints but retain the full system, this approach reduces the total number of atoms, leading to substantially lower resource consumption and runtime efficiency, especially for protein–ligand interactions involving large supramolecular structures.

## Supplementary Material



## Data Availability

The generated data are presented in the Supporting Information file. The codes used in the study are available at https://github.com/kurkcuoglulevitaslab/mcg_ANM.

## Notes

MD simulations (Table S1); XP docking scores across holo, apo, truncated, and ANM-derived conformers (Table S2); binding free energy calculations via MM-GBSA (Table S3); the structural comparison of MCG ANM and AF2-generated conformers (Table S4); comparisons of experimental and ANM-predicted B-factors for *Gg*TIM and *Pf*TIM (Figures S1 and S9); molecular docking studies for PGH and DHAP with *Gg*TIM presenting crystal poses, redocking validations, and key interactions across subunits and structural states (Figures S2 and S3); restraint schemes used to stabilize truncated structures during simulations (Figure S4), alongside contact profiles, secondary structure changes, and ANM mode overlap comparisons across conditions and replicas (Figure S5); ligand–protein interactions observed throughout MD simulations for *Gg*TIM-DHAP and *Pf*TIM-3PG, including intact and ANM-derived conformers (Figures S6–S8, S11 and S12); 3PG binding at the *Gg*TIM dimer interface across native and deformed conformers (Figure S14); the MCG ANM and AF2-generated conformers (Figure S15) (PDF)
