# Supplementary material for: A Computationally Efficient Method to Generate Plausible Conformers for Ensemble Docking and Binding Free Energy Calculations
Source: J Chem Inf Model. 2025 Jul 23;65(15):8137–57. doi: 10.1021/acs.jcim.5c00431 (PMC12344705; doi:10.1021/acs.jcim.5c00431)
Supplement: Supplementary file 1 [file ci5c00431_si_001.pdf]

SUPPORTING INFORMATION

A Computationally Efficient Method to Generate Plausible Conformers for Ensemble Docking and Binding Free Energy Calculations

Ö. Zeynep Güner Yılmaz[1], Pemra Doruker[2ɫ], and Ozge Kurkcuoglu[1]*

[1]Department of Chemical Engineering, Istanbul Technical University, Istanbul, Turkey

[2]Department of Computational and Systems Biology, School of Medicine, University of Pittsburgh, Pittsburgh, PA 15213, USA

*Corresponding Author: Ozge Kurkcuoglu, olevitas@itu.edu.tr

ɫCurrent address: Allotar Therapeutics, USA

Table S1. Details of the MD simulations

System name		# atoms	# water molecules	# ions	Duration
*Gg*TIM_Caseref	Intact, subunit A&B	49,474	13,468	39 Na^+<br>39 Cl^-	2×100ns
*Gg*TIM_Case1	Truncated structure	25,160	7,119	22 Na^+<br>20 Cl^-	2×100ns
*Gg*TIM_Case2	Truncated structure	25,160	7,119	22 Na^+<br>20 Cl^-	2×100ns
*Gg*TIM_Case3	Truncated structure	25,160	7,119	22 Na^+<br>20 Cl^-	2×100ns
*Gg*TIM	Intact, subunit A&B	49,474	13,468	39 Na^+<br>39 Cl^-	3×300 ns
*Gg*TIM (subunit B)	Truncated structure	25,160	7,119	22 Na^+<br>20 Cl^-	3×300 ns
*Gg*TIM_m1a	Truncated conformer	25,873	7,377	26 Na^+<br>20 Cl^-	3×300 ns
*Gg*TIM_m1b	Truncated conformer	24,910	7,056	26 Na^+<br>20 Cl^-	3×300 ns
*Gg*TIM_m2a	Truncated conformer	25,828	7,362	26 Na^+<br>20 Cl^-	3×300 ns
*Gg*TIM_m2b	Truncated conformer	24,865	7,041	26 Na^+<br>20 Cl^-	3×300 ns
*Gg*TIM_m3a	Truncated conformer	25,693	7,317	26 Na^+<br>20 Cl^-	3×300 ns
*Gg*TIM_m3b	Truncated conformer	25,717	7,325	26 Na^+<br>20 Cl^-	3×300 ns
*Pf*TIM	Intact, subunit A&B	40,847	10,975	43 Na^+<br>31 Cl^-	3×300 ns
*Pf*TIM_truncated	Truncated structure	31,669	9,063	34 Na^+<br>25 Cl^-	3×300 ns
*Pf*TIM_m1a	Truncated conformer	35,283	10,265	38 Na^+<br>29 Cl^-	3×300 ns
*Pf*TIM_m1b	Truncated conformer	33,725	9,747	36 Na^+<br>27 Cl^-	3×300 ns
*Pf*TIM_m2a	Truncated conformer	34,960	10,501	37 Na^+<br>28 Cl^-	3×300 ns
*Pf*TIM_m2b	Truncated conformer	34,180	10,241	37 Na^+<br>28 Cl^-	3×300 ns
*Pf*TIM_m3a	Truncated conformer	34,813	10,109	37 Na^+<br>28 Cl^-	3×300 ns
*Pf*TIM_m3b	Truncated conformer	34,333	9,949	37 Na^+<br>28 Cl^-	3×300 ns

Table S2. XP docking scores (kcal/mol) obtained from Schrödinger Glide for ligand-protein interactions across three systems: (1) *Gg*TIM catalytic site with DHAP, (2) *Pf*TIM dimer interface with 3PG, and (3) *Gg*TIM dimer interface with 3PG. Results are shown for the holo, apo, truncated, and ANM-derived conformers (with deformation RMSD of 1.0 and 1.5 Å).

**System (1)**	**XP Score (kcal/mol)**	**System (2)**	**XP Score (kcal/mol)**	**System (3)**	**XP Score (kcal/mol)**
Holo *Gg*TIM (subunit A)	-7.58	Holo *Pf*TIM (subunit A)	-6.23	Holo *Pf*TIM (subunit A)	-6.23
Holo *Gg*TIM (subunit B)	-7.43	Holo *Pf*TIM (subunit B)	-6.42	Holo *Pf*TIM (subunit B)	-6.42
Holo *Gg*TIM (subunit A)	-7.29	Apo *Pf*TIM (subunit A)	-2.15	Apo *Gg*TIM (subunit A)	-2.93
Holo *Gg*TIM (subunit B)	-6.56	Apo *Pf*TIM (subunit B)	-6.02	Apo *Gg*TIM (subunit B)	-3.07
Apo *Gg*TIM (subunit A)	-3.91	Apo *Pf*TIM (truncated)	-5.59	Apo *Gg*TIM (truncated)	-3.86
Apo *Gg*TIM (subunit B)	-4.46	*Pf*TIM_m1a	-5.89	Apo *Gg*TIM_m1a	-2.81
Apo *Gg*TIM (truncated, subunit B)	-5.23	*Pf*TIM_m1b	-5.58	Apo *Gg*TIM_m1b	-2.76
*Gg*TIM_m1a	-4.83	*Pf*TIM_m2a	-5.99	Apo *Gg*TIM_m2a	-3.21
*Gg*TIM_m1b	-5.09	*Pf*TIM_m2b	-5.21	Apo *Gg*TIM_m2b	-3.41
*Gg*TIM_m2a	-4.80	*Pf*TIM_m3a	-5.50	Apo *Gg*TIM_m3a	-3.77
*Gg*TIM_m2b	-4.59	*Pf*TIM_m3b	-5.85	Apo *Gg*TIM_m3b	-3.77
*Gg*TIM_m3a	-4.55	*Pf*TIM_m1a	-5.39		
*Gg*TIM_m3b	-4.91	*Pf*TIM_m1b	-5.24		
*Gg*TIM_m1a	-4.69	*Pf*TIM_m2a	-3.61		
*Gg*TIM_m1b	-5.08	*Pf*TIM_m2b	-3.14		
*Gg*TIM_m2a	-4.77	*Pf*TIM_m3a	-4.09		
*Gg*TIM_m2b	-5.44	*Pf*TIM_m3b	-2.72		
*Gg*TIM_m3a	-3.90				
*Gg*TIM_m3b	-4.77				

Table S3. ΔG_{bind} (kcal/mol) values calculated with MM-GBSA approach.

**System**		**ΔG_{bind} (kcal/mol)**						
		**50 ns**	**100 ns**	**200 ns**	**300 ns**			
		average*	average	average	Run 1	Run 2	Run 3	average
*Gg*TIM	Subunit A	-28.24 ± 4.54	-26.78 ±5.32	-27.19 ±3.44	-35.41±3.64	-27.67±5.19	-14.87±7.89	-25.98 ±5.47
*Gg*TIM	Subunit B	-32.03 ±1.64	-31.55 ±0.16	-29.41 ±1.90	-30.90±3.88	-30.47±2.85	-35.22±4.46	-30.69 ±0.30
*Gg*TIM	Truncated	-28.99 ±1.50	-28.68 ±1.46	-28.04 ±1.36	-25.50±3.93	-28.08±3.41	-26.38±3.70	-28.08 ±1.31
*Gg*TIM_m1a	Truncated	-25.20 ±0.54	-25.29 ±0.12	-26.13 ±0.98	-26.59±4.06	-25.79±2.83	-26.86±4.14	-26.41 ±0.56
*Gg*TIM_m1b	Truncated	-30.76 ±1.03	-30.59 ±1.23	-30.16 ±1.05	-31.31±2.50	-29.40±4.28	-30.46±3.37	-30.39 ±0.78
*Gg*TIM_m2a	Truncated	-32.70 ±2.84	-32.46 ±2.83	-32.16 ±2.89	-32.75±4.94	-32.37±4.48	-28.66±3.87	-31.26 ±2.26
*Gg*TIM_m2b	Truncated	-33.14 ±1.37	-32.52 ±2.26	-33.01 ±1.73	-33.91±2.46	-34.04±2.27	-32.12±4.34	-33.36 ±1.07
*Gg*TIM_m3a	Truncated	-28.96 ±1.24	-29.26 ±0.76	-29.67 ±0.37	-29.82±4.08	-30.28±2.75	-29.46±3.31	-29.85 ±0.41
*Gg*TIM_m3b	Truncated	-27.61 ±1.14	-27.30 ±0.86	-25.86 ±1.07	-26.37±5.44	-25.49±5.09	-27.34±4.78	-26.40 ±0.93
*Pf*TIM	Subunit A	-32.67±0.68	-33.13±1.36	-32.54±2.84	-38.95±7.85	-30.51±5.51	-35.51± 7.23	-33.01±3.53
*Pf*TIM	Subunit B	-37.07±5.94	-36.84±5.44	-35.35±5.87	-35.34±6.37	-35.70±6.00	-35.87±6.67	-36.33±6.10
*Pf*TIM	Truncated	-35.54±0.20	-35.71±0.11	-35.64±0.30	-40.43±3.07	-39.87±2.52	-40.49±2.74	-35.64±0.27
*Pf*TIM_m1a	Truncated	-40.01±0.48	-40.24± 0.43	-40.29±0.34	-46.31±2.56	-45.41±2.52	-44.95±2.57	-40.26±0.34
*Pf*TIM_m1b	Truncated	-44.92±0.18	-45.35±0.56	-45.50±0.53	-44.09±2.27	-43.71±2.22	-43.89±2.22	-45.56±0.69
*Pf*TIM_m2a	Truncated	-43.89±0.17	-44.03±0.14	-43.93±0.30	-47.01±2.84	-47.16±2.75	-47.23±2.61	-43.90±0.27
*Pf*TIM_m2b	Truncated	-47.14±0.46	-47.23±0.19	-47.28±0.06	-43.35±2.33	-44.08±2.21	-45.06±2.28	-47.13±0.11
*Pf*TIM_m3a	Truncated	-44.20±1.08	-43.96±0.96	-44.10±0.90	-34.74±2.53	-31.37±2.91	-32.98±2.41	-44.16±0.86
*Pf*TIM_m3b	Truncated	-33.01±3.09	-33.54±3.98	-33.40±3.82	-39.37±3.27	-38.84±2.88	-40.77±3.22	-33.70±4.38

*average over three replicates

Table S4. Structural comparison of AlphaFold2- and MCG-ANM–generated *Gg*TIM conformers using loop 6 (residues 168–178) RMSD, and inter-residue Cα–Cα distances between Gly171 (loop 6) and Gly210 and Asn216 (loop 7). Distances in dimers are reported for each chain if different. Experimental apo (PDB ID: 8TIM) and holo (PDB ID: 1TPH) structures are included for reference. pLDDT and pTM scores indicate model confidence, where available.

Model	Source	Structure	Loop 6 RMSD (Å)*	Distance between G171-N216 (Å)	Distance between G171-G210 (Å)	pLDDT**	pTM
Apo, open loop 6	PDB ID: 8TIM	Dimer	-	16.3 (chA) / 18.2 (chB)	9.6 (chA) / 11.7 (chB)	-	-
Holo, closed loop 6	PDB ID: 1TPH	Dimer	-	14.1 (chA) / 14.2 (chB)	6.4	-	-
m1a	MCG-ANM	Dimer	0.95	18.0	13.8	-	-
m1b	MCG-ANM	Dimer	0.95	19.3	13.6	-	-
m2a	MCG-ANM	Dimer	0.95	19.4	13.6	-	-
m2b	MCG-ANM	Dimer	0.95	19.3	13.6	-	-
m3a	MCG-ANM	Dimer	1.01	19.7	13.9	-	-
m3b	MCG-ANM	Dimer	0.95	19.0	13.6	-	-
af1	AF2 Server	Dimer	1.30	14.3	6.6	> 90	0.97
af2	AF2 Server	Dimer	1.32	14.4	6.6	> 90	0.97
af3	AF2 Server	Dimer	1.31	14.4	6.6	> 90	0.97
af4	AF2 Server	Dimer (no template)	1.18	15.2	9.2	> 90	0.97
af5	AF2 Server	Dimer (no template)	1.01	16.4	9.7	> 90	0.95
af6	AF2 Server	Dimer (no template)	0.95	16.5	10.0	> 90	0.95
af7	AF2 Server	Monomer	1.28	14.5	7.7	> 90	0.95
af8	AF2 Server	Monomer	1.29	14.6	7.8	> 90	0.97
af9	AF2 Server	Monomer	1.30	14.5	7.7	> 90	0.97
af10	AF2 Server	Monomer (no template)	1.35	14.9	8.3	> 90	0.97
af11	AF2 Server	Monomer (no template)	1.38	14.8	8.2	> 90	0.97
af12	AF2 Server	Monomer (no template)	1.36	14.8	8.2	> 90	0.97
cf1	ColabFold	Monomer	0.98	15.5	8.7	96.7	0.94
cf2	ColabFold	Monomer	1.20	16.7	9.5	96.5	0.93
cf3	ColabFold	Monomer	0.90	15.8	8.6	93.2	0.92
cf4	ColabFold	Monomer	0.96	15.2	8.8	92.9	0.92
cf5	ColabFold	Monomer	1.05	15.3	8.4	91.8	0.92
cf6	ColabFold	Monomer	1.03	15.0	8.4	96.4	0.94

*Loop 6 RMSD was calculated with respect to the apo form (PDB ID: 8TIM). **AF2 server gives the pLDDT scores with thresholds. pLDDT scores from ColabFold are reported as average values.

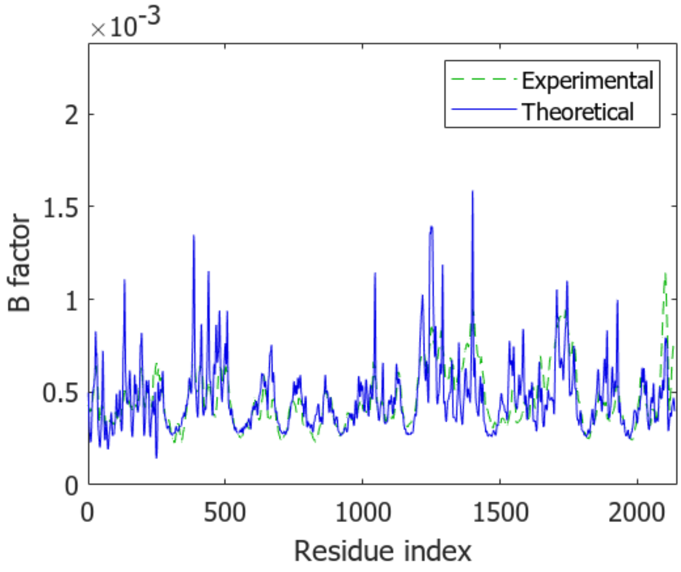

Figure S1. Experimental and ANM-calculated B-factors for *Gg*TIM.

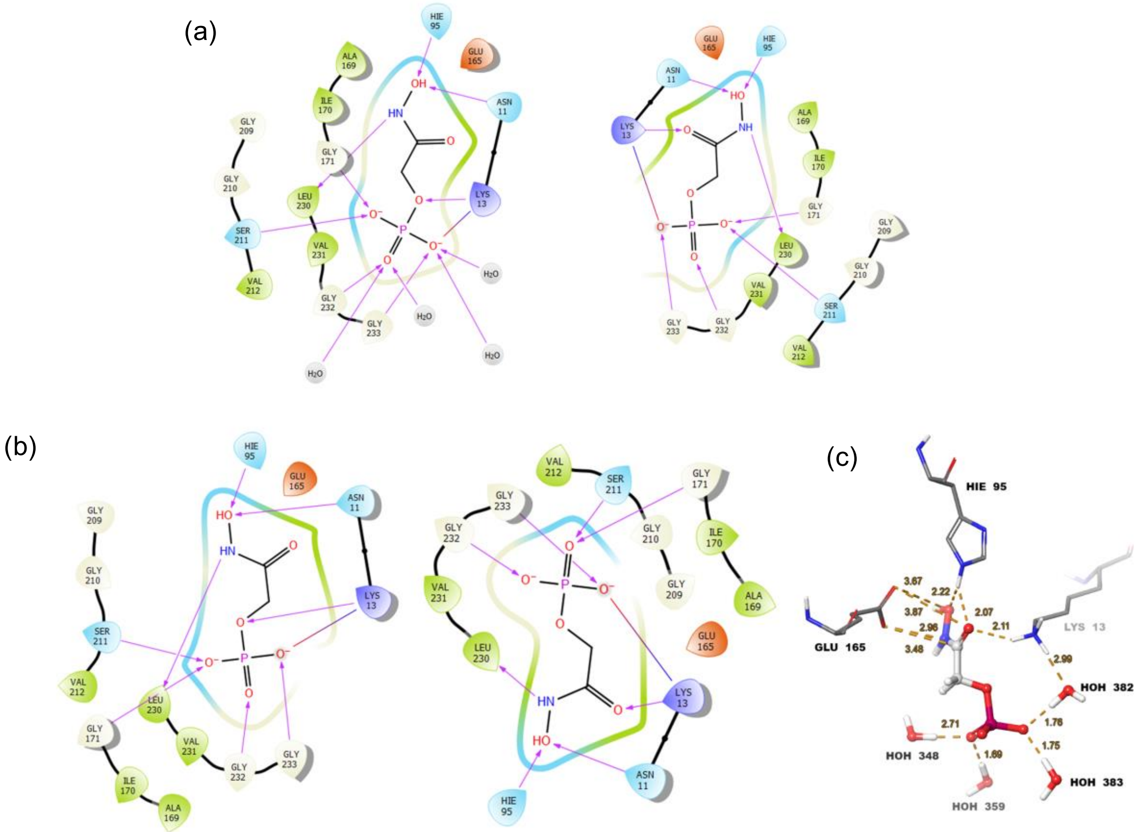

Figure S2. Molecular docking studies of *Gg*TIM (PDB ID: 1TPH) with PGH. (a) The crystal pose of PGH in two subunits A and B of *Gg*TIM structure. (b) Interactions between PGH and *Gg*TIM after re-docking. (c) Distances between the key residues and PGH after re-docking.

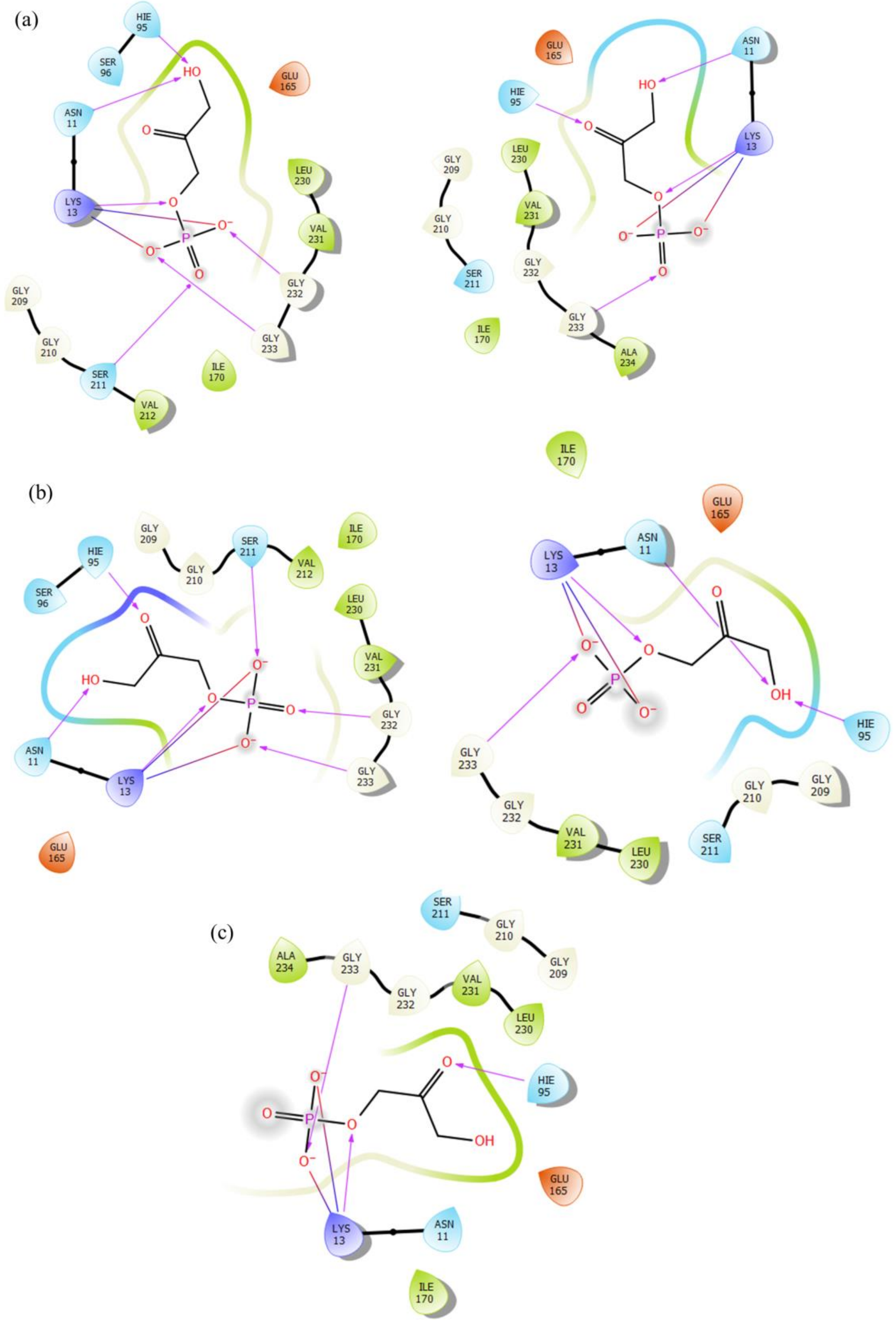

Figure S3. (a) Interactions between DHAP and *Gg*TIM (PDB ID: 1TPH) after molecular docking in subunit A and B. Interactions between DHAP and the apo *Gg*TIM (PDB ID: 8TIM) in (b) subunit A and B, (d) truncated structure according to molecular docking calculations.

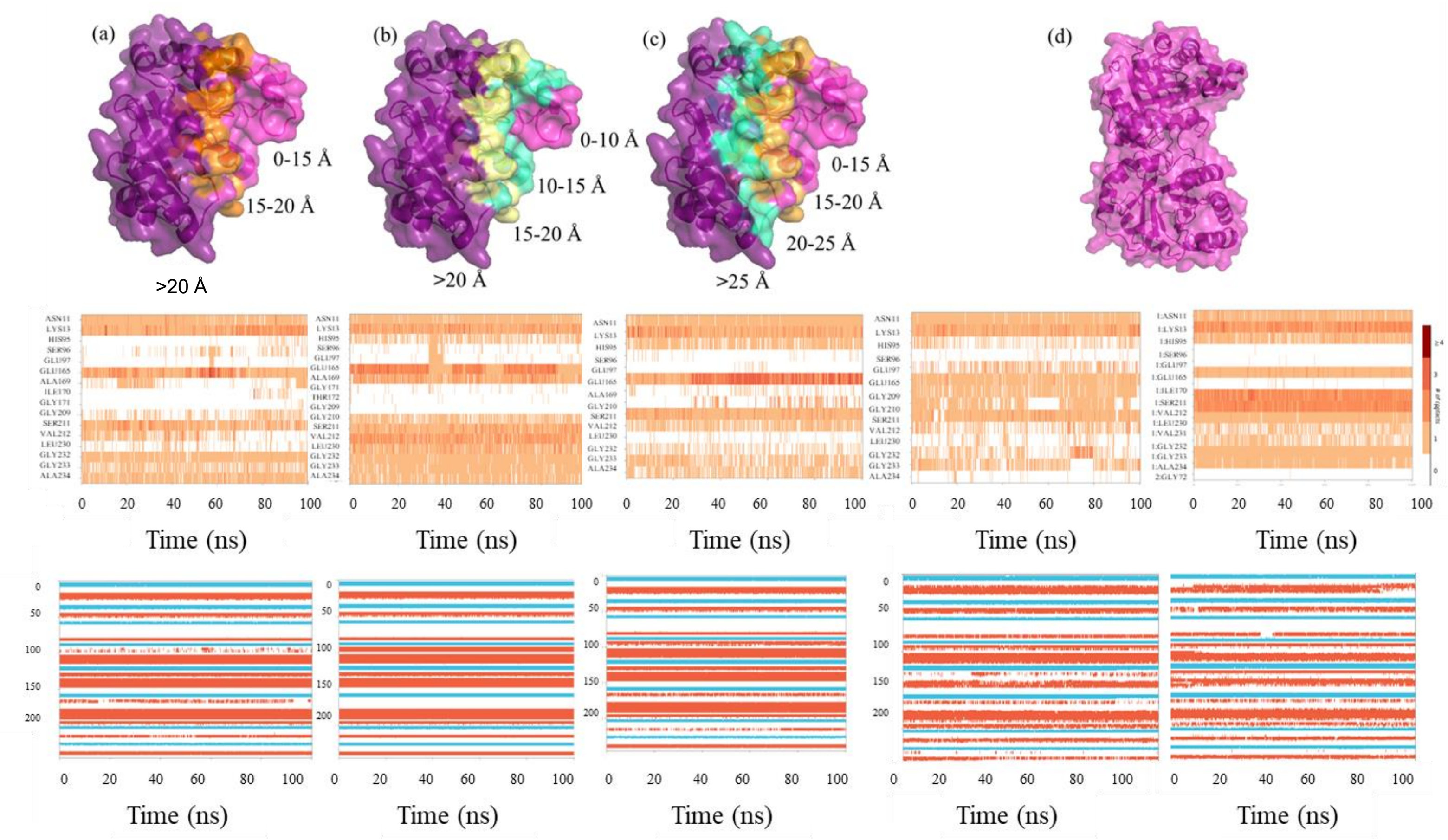

Figure S4. Three different cases tested to prevent the disintegration of the truncated structure during MD simulations. (a) Case 1: No force applied within 15 Å (magenta), 10 kcal/mol. Å^2 force applied within 15-20 Å (orange), and 50 kcal/mol. Å^2 force applied beyond 20 Å (purple). (b) Case 2: No force applied within 10 Å (magenta), 25 kcal/mol.Å^2 force applied within 10-15 Å (cyan), 35 kcal/mol.Å^2 force applied within 15-20 Å (yellow), and 50 kcal/mol.Å^2 force applied beyond 20 Å (purple). (c) Case 3: No force applied within 15 Å (magenta), 10 kcal/mol.Å^2 force applied within 15-20 Å (orange), and 25 kcal/mol.Å^2 force applied beyond 20 Å (cyan). (d) Unrestrained full holo-TIM structure for comparison. Below each panel, graphs depict ligand interactions (middle panel) and changes in secondary structure (red for α-helix, blue for β-strand, lowest panel) throughout the simulation.

(a)

	Intact ChA (R1)	Intact ChA (R2)	Intact ChB (R1)	Intact ChB (R2)	Case 1 (R1)	Case 1 (R2)	Case 2 (R1)	Case 2 (R2)	Case 3 (R1)	Case 3 (R2)
Asn11	95	92	94	25	95	93	94	99	96	52
Lys13	98	95	93	74	95		85	97	99	98
His95	57	85	33	60			60	86	77	49
Glu165	96	100		39	97	89	92	100	84	53
Ser211	88	82	79	28	54	93	92	100	96	18

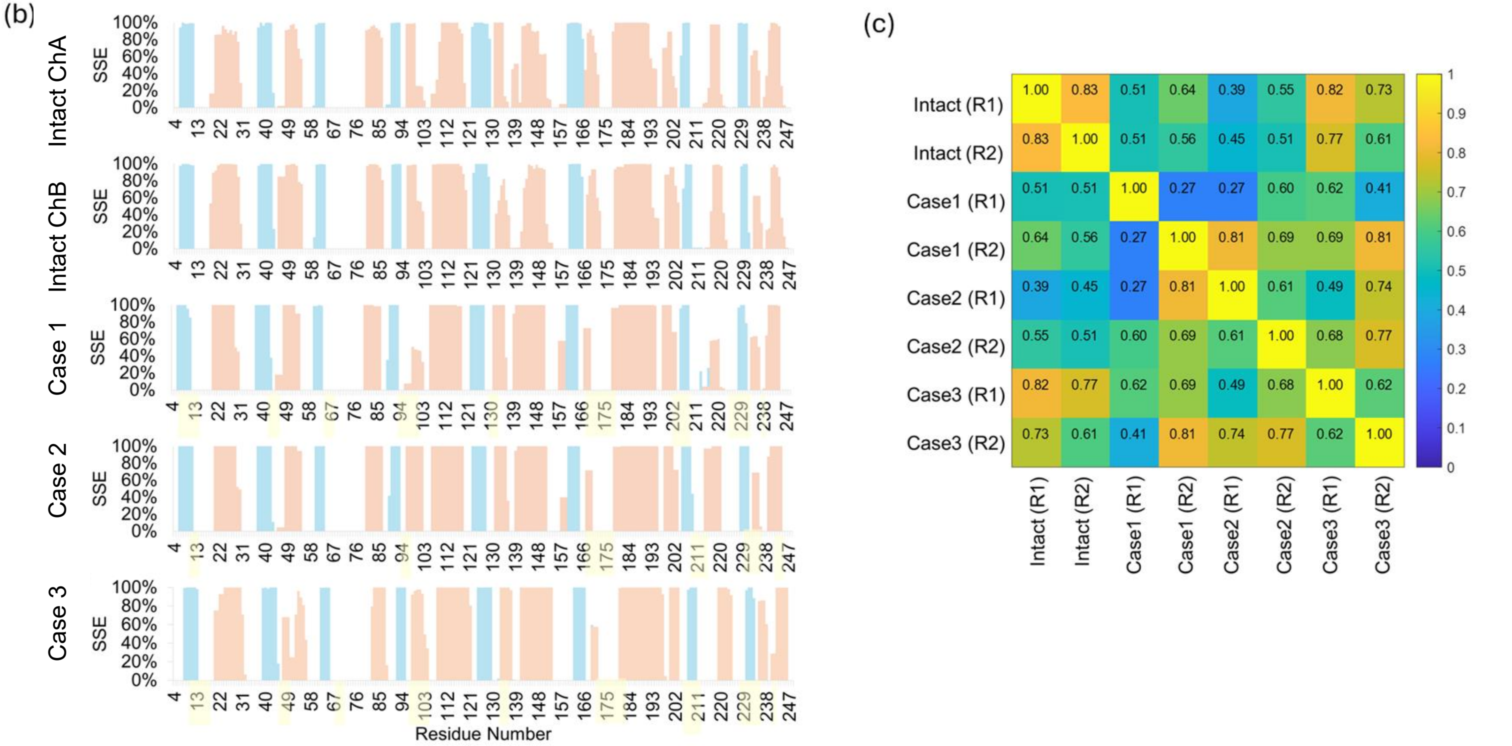

Figure S5. (a) Percentages of interactions between the ligand DHAP and the active-site residues (Asn11, Lys13, His95, Glu165, Ser211) during the simulations of the investigated systems and their replicas (Intact and Cases 1–3, replicas R1 and R2). (b) Secondary structure element (SSE) occupancy profiles for all systems, with blue and orange bars indicating α-helices and β-strands, respectively. Yellow shades on the residue indices mark the residues that are kept flexible. (c) Correlation coefficients between the root mean squared fluctuations of the residues that are kept flexible during the simulations.

(a)

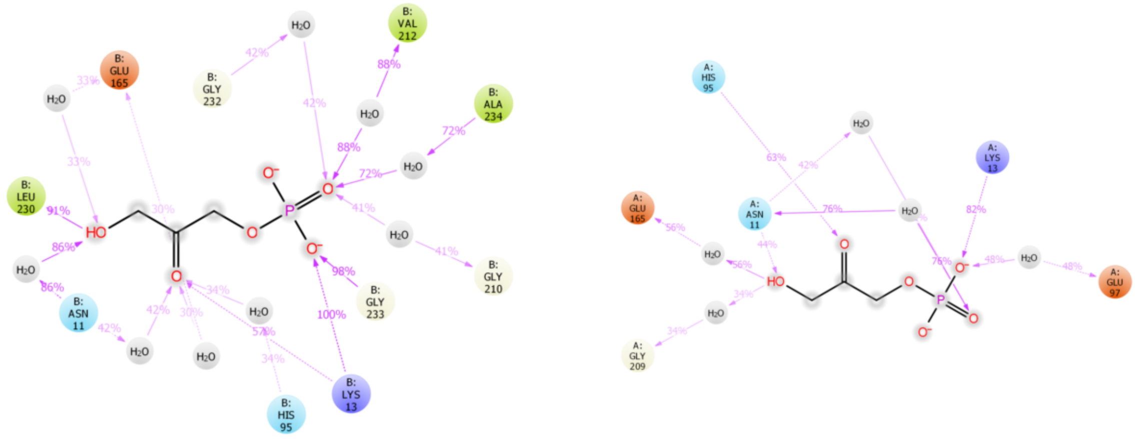

(b)

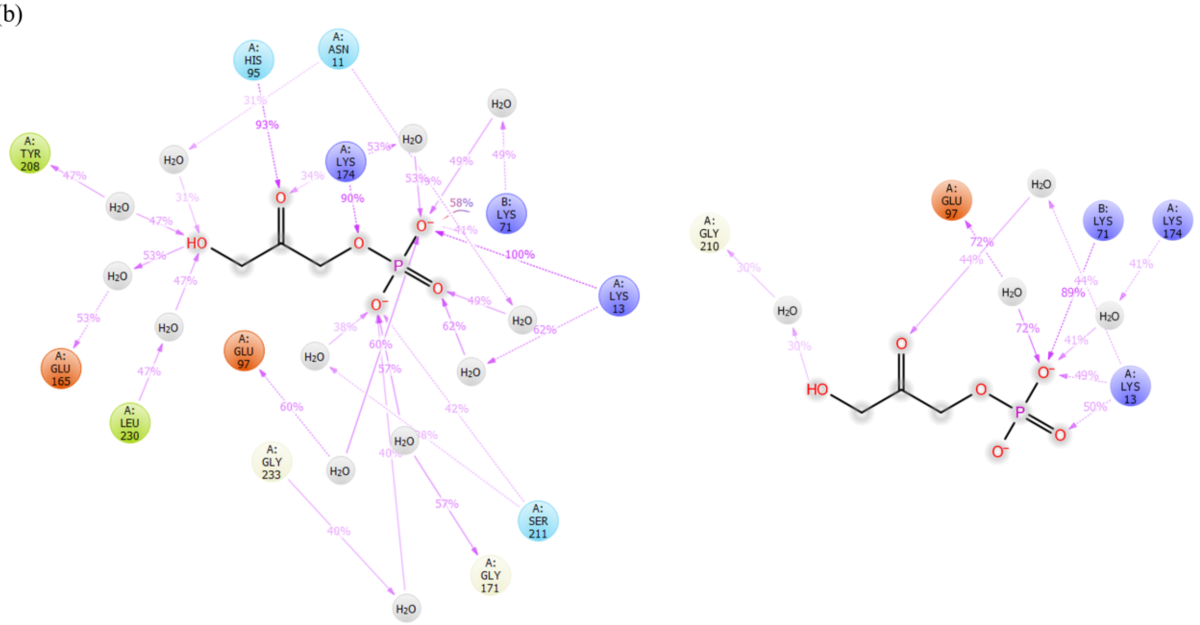

Figure S6. Ligand-protein interactions observed throughout MD simulations of intact *Gg*TIM-DHAP structures for subunits A and B along (a) the second replica and (b) the third replica.

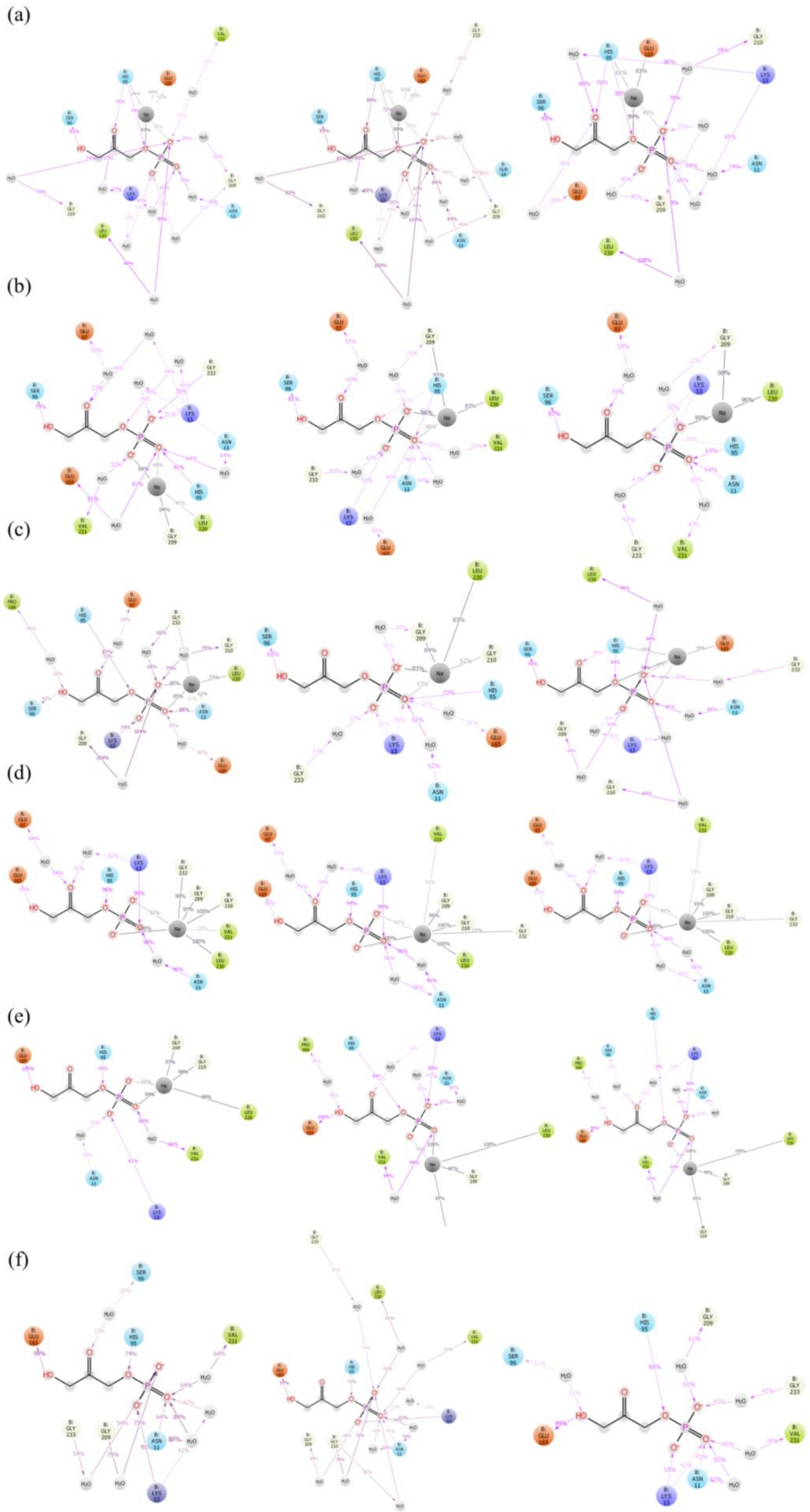

Figure S7. Protein-ligand interactions throughout MD simulations of *Gg*TIM (a) m1a, (b) m1b, (c) m2a, (d) m2b, (e) m3a, (f) m3b. Interactions for three replicates are displayed in order from left to right.

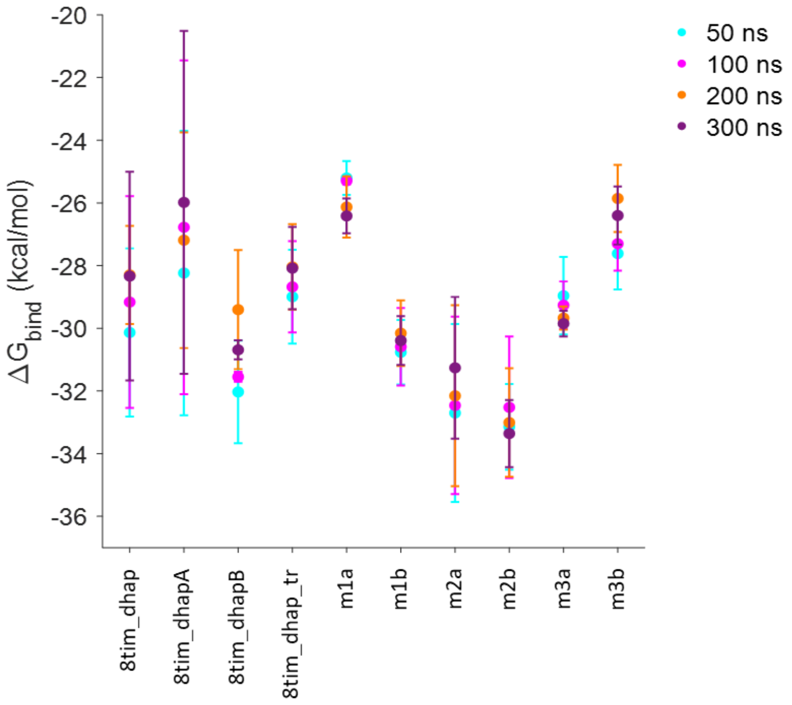

Figure S8. Binding free energy (ΔG_{bind}) calculated using MM-GBSA across different time intervals: first 50, first 100, first 200, and 300 ns for truncated TIM structures. R1 (cyan), R2 (magenta) and R3 (orange) represent three replicate simulations.

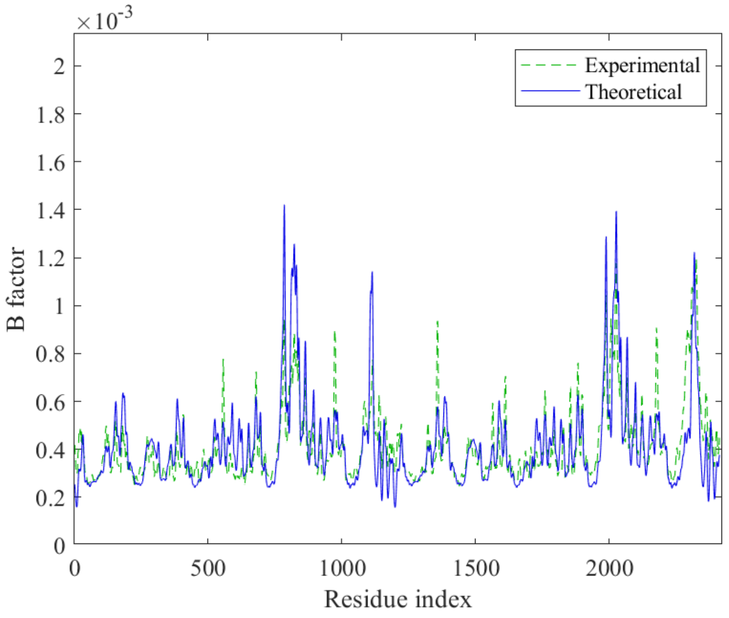

Figure S9. Experimental and ANM-calculated B-factors for *Pf*TIM.

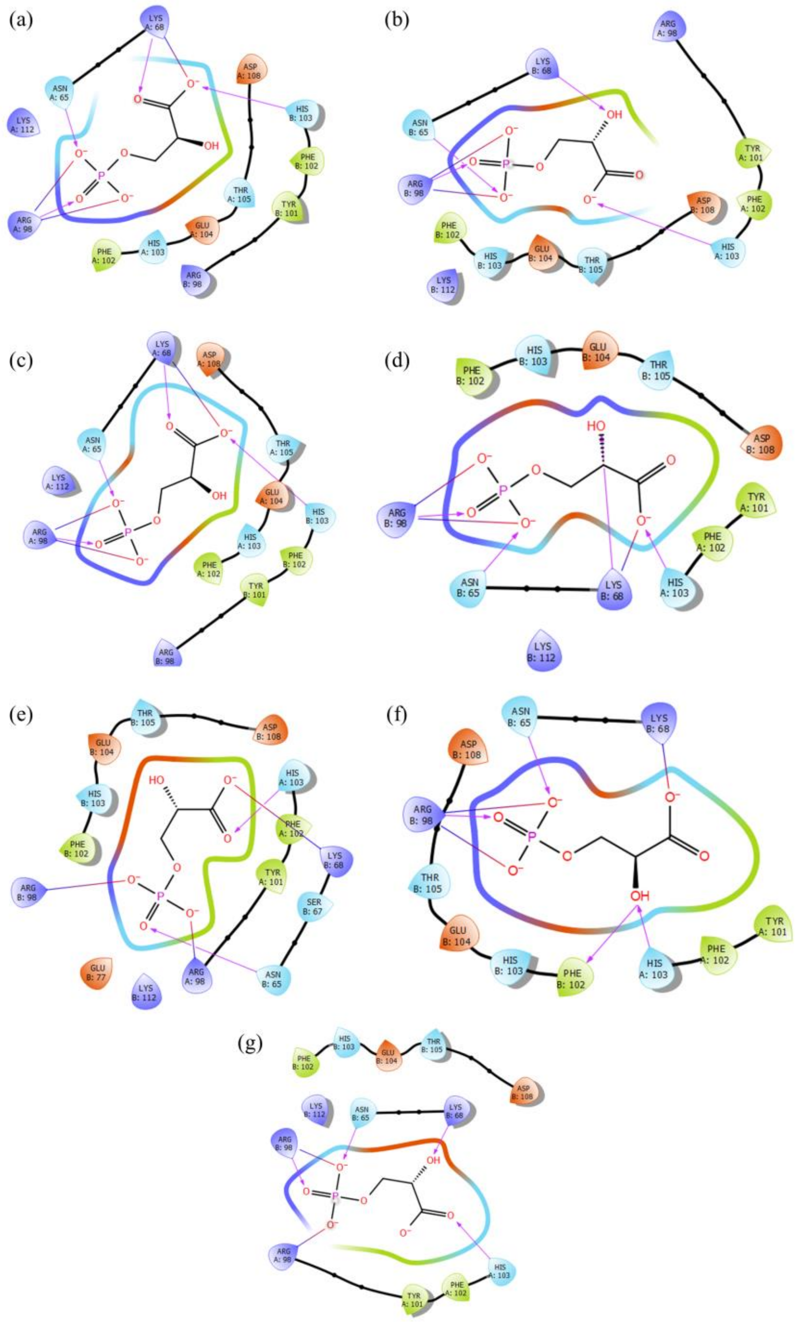

Figure S10. Interaction diagrams of (a) chain A and (b) chain B in the crystal structure of holo *Pf*TIM, and (c) chain A and (d) chain B after the validation docking. (e) and (f) show the results of docking into the apo *Pf*TIM structure in both chains, while (g) shows the interactions of the ligand in the truncated structure using the central region in chain B.

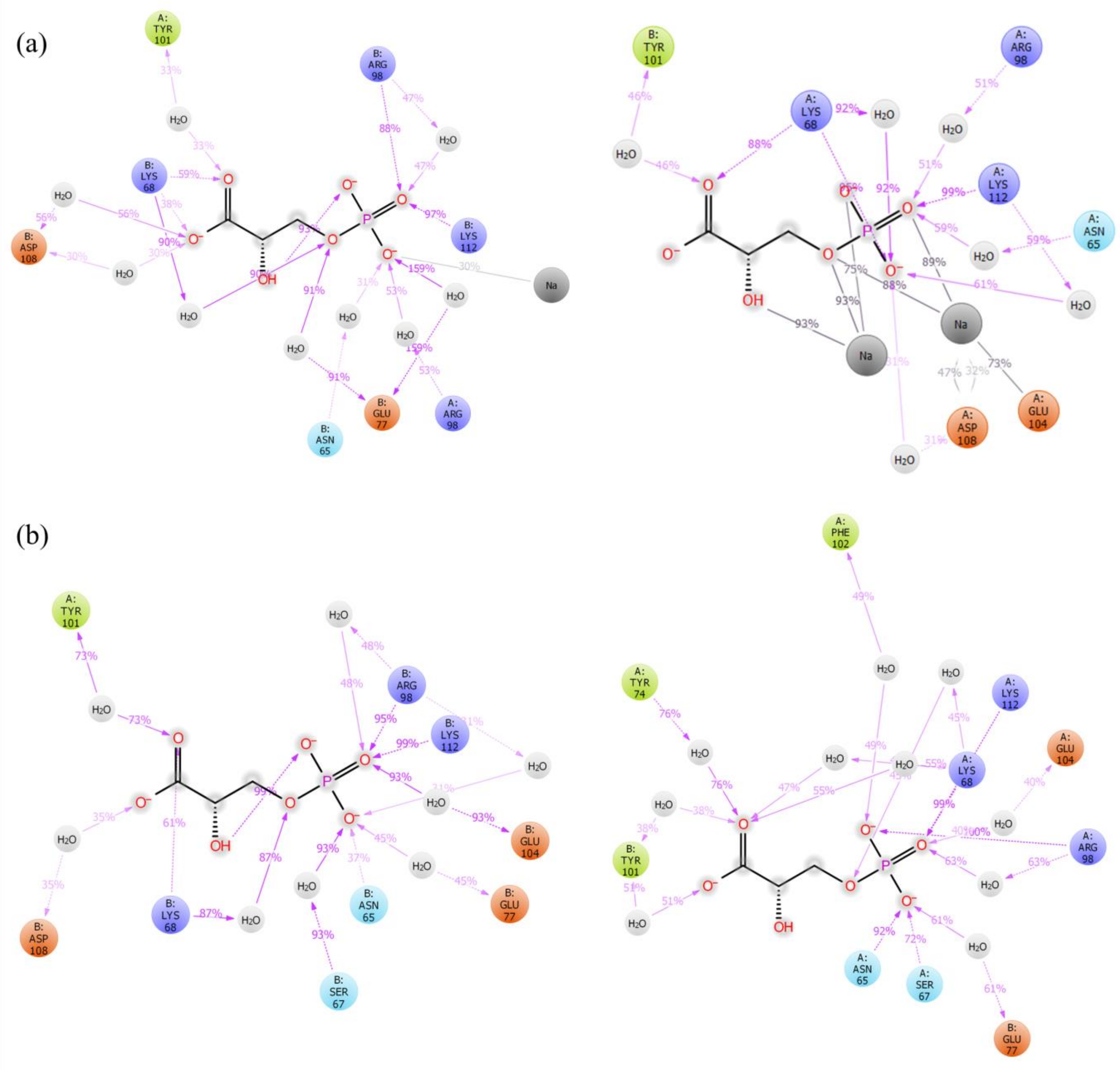

Figure S11. Ligand-protein interactions observed throughout MD simulations of intact *Pf*TIM-3PG structures for subunits A and B along (a) the second replica and (b) the third replica.

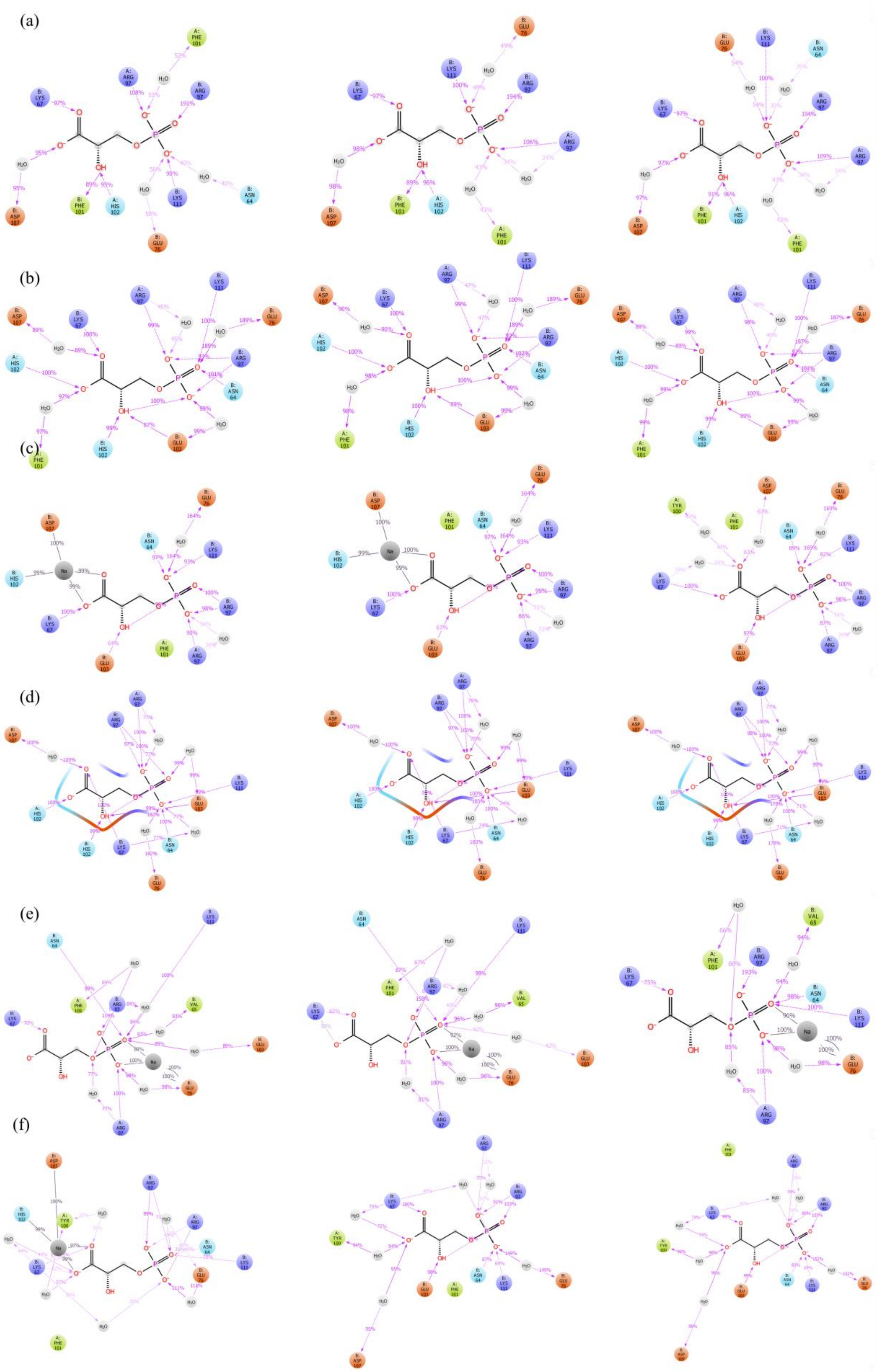

Figure S12. Protein-ligand interactions throughout MD simulations of *Pf*TIM (a) m1a, (b) m1b, (c) m2a, (d) m2b, (e) m3a, (f) m3b. Interactions for three replicates are displayed in order from left to right.

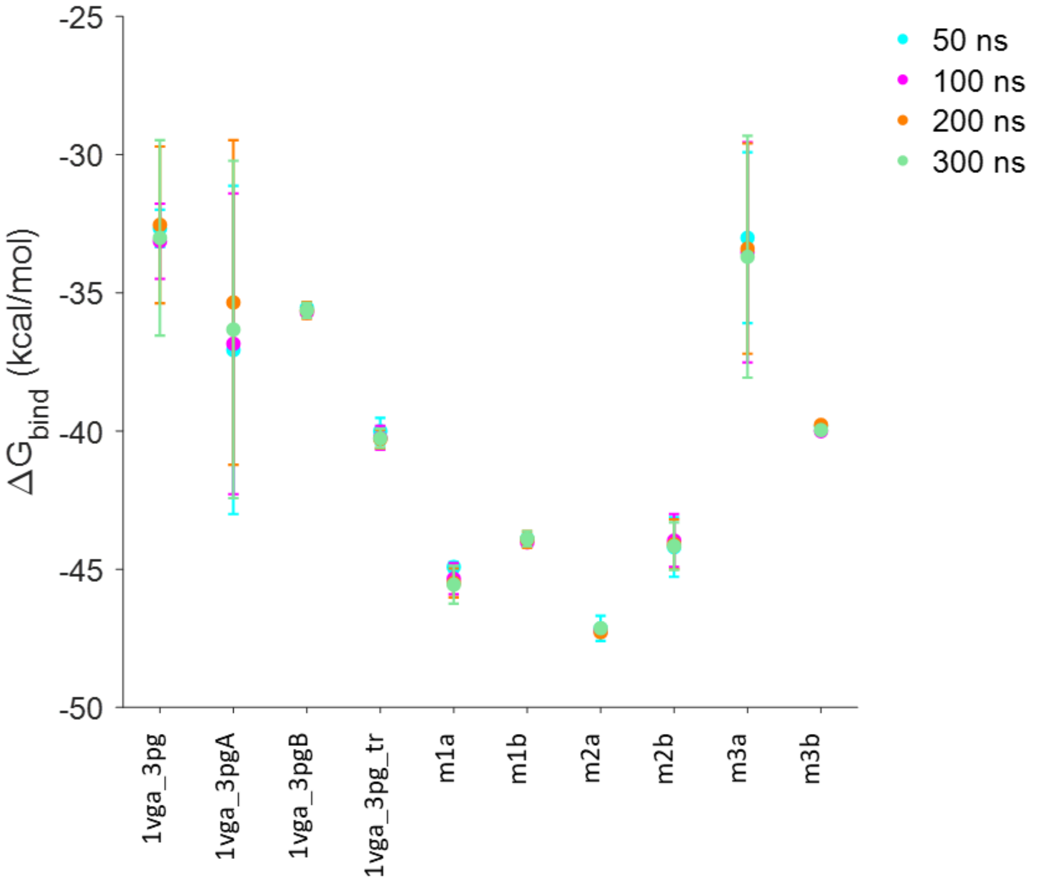

Figure S13. ΔG_{bind} calculated using MM-GBSA across different time intervals: first 50, first 100, first 200, and 300 ns for truncated TIM structures. R1 (cyan), R2 (magenta) and R3 (orange) represent three replicate simulations.

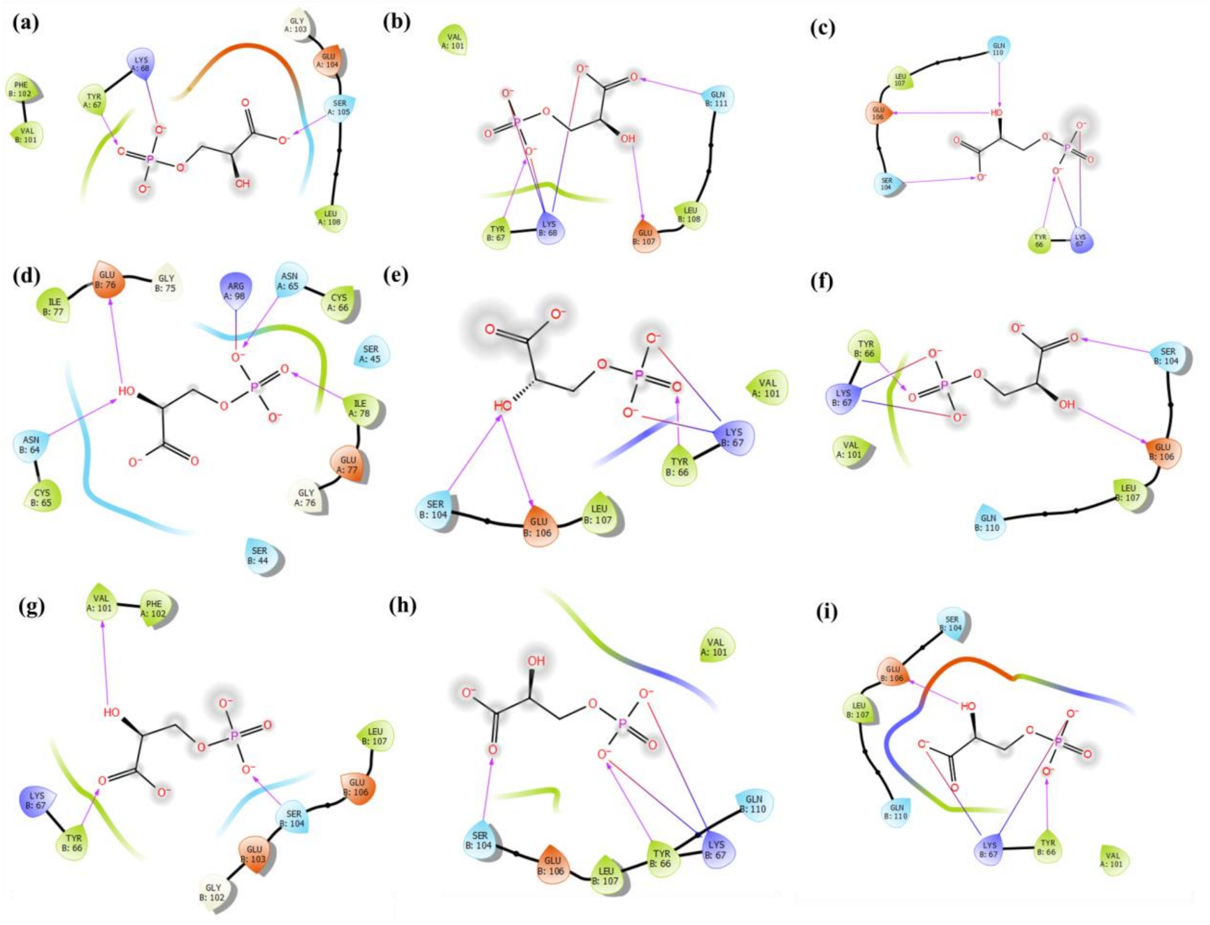

Figure S14. Interaction diagrams of 3PG ligand with different conformations of the *Gg*TIM dimer interface: (a) *Gg*TIM intact chain A, (b) *Gg*TIM intact chain B, (c) *Gg*TIM truncated chain B, (d) *Gg*TIM_m1a, (e) *Gg*TIM_m2a, (f) *Gg*TIM_m3a, (g) *Gg*TIM_m1b, (h) *Gg*TIM_m2b, (i) *Gg*TIM_m3b.

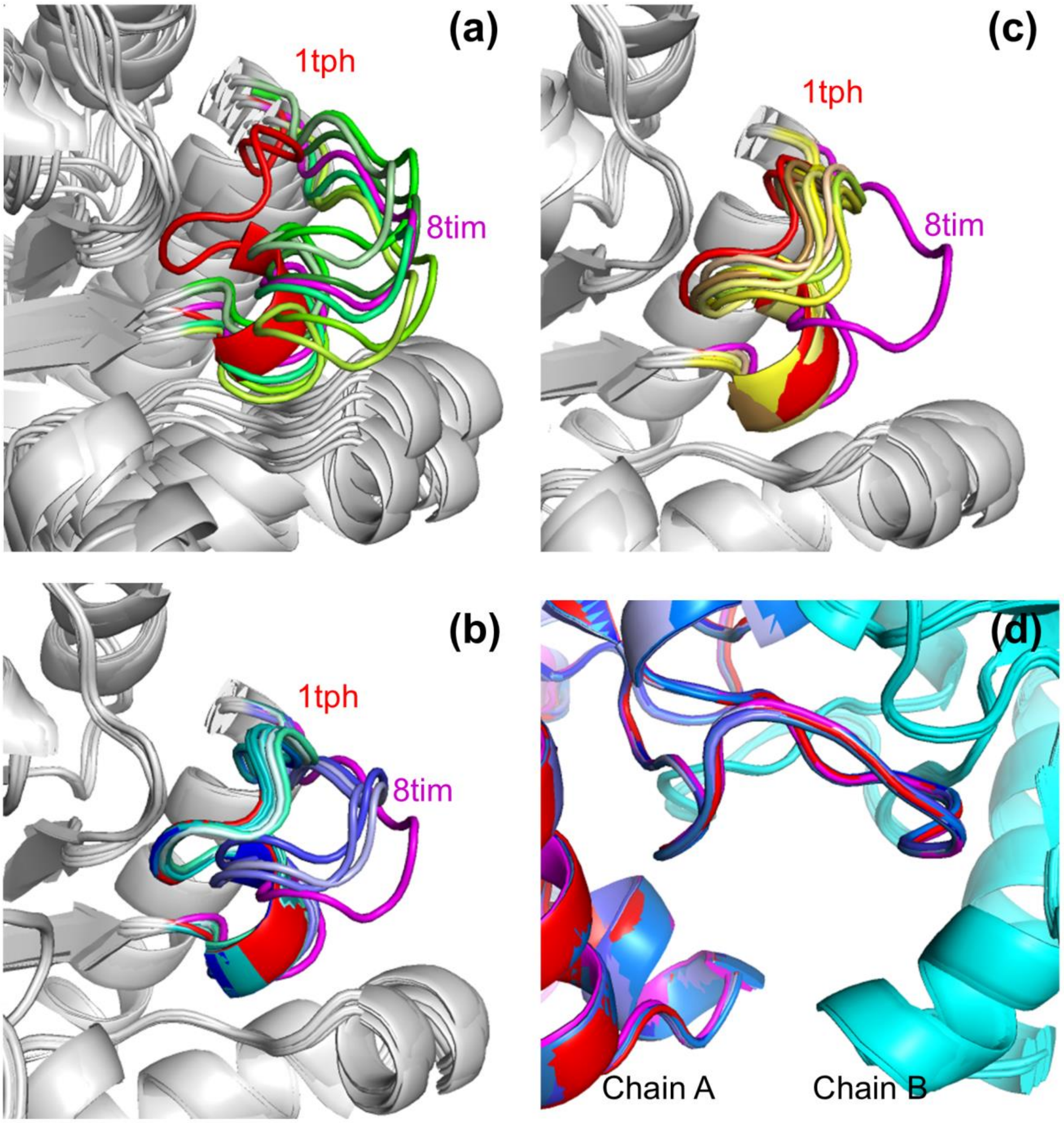

Figure S15. Structural alignment of *Gg*TIM conformers showing the loop 6 generated with (a) MCG-ANM, (b) AF2, (c) ColabFold with varying MSA depths. (d) AF2-generated dimer models showing the dimer interface, where only chains A are colored to highlight the conformers. The reference structure in the alignment is PDB ID: 8TIM. The open (in magenta) and closed (in red) conformations of loop 6 are shown by the apo (PDB ID: 8TIM) and holo (PDB ID: 1TPH) structures, respectively.
